# Taphonomic and spatial analyses from the Early Pleistocene site of Venta Micena 4 (Orce, Guadix-Baza Basin, southern Spain)

**DOI:** 10.1038/s41598-021-93261-1

**Published:** 2021-07-07

**Authors:** Carmen Luzón, Jose Yravedra, Lloyd A. Courtenay, Juha Saarinen, Hugues-Alexandre Blain, Daniel DeMiguel, Suvi Viranta, Beatriz Azanza, Juan José Rodríguez-Alba, Darío Herranz-Rodrigo, Alexia Serrano-Ramos, Jose A. Solano, Oriol Oms, Jordi Agustí, Mikael Fortelius, Juan Manuel Jiménez-Arenas

**Affiliations:** 1 Departamento de Prehistoria y Arqueología, Universidad de Granada, Granada, Spain; 2 Departamento de Prehistoria, Universidad Complutense, Madrid, Spain; 3 C.A.I. Arqueometría, Universidad Complutense, Madrid, Spain; 4 Departamento de Ingeniería Cartográfica y del Terreno, Escuela Politécnica Superior de Ávila, Universidad de Salamanca, Ávila, Spain; 5 Department of Geosciences and Geology, University of Helsinki, Helsinki, Finland; 6 Institut Català de Paleoecologia Humana i Evolució Social (IPHES-CERCA), Tarragona, Spain; 7 Departament d’Història i Història de l’Art, Universitat Rovira i Virgili, Tarragona, Spain; 8 ARAID/Departmento de Ciencias de la Tierra (Paleontología), Universidad de Zaragoza, Zaragoza, Spain; 9 Institut Català Paleontologia M. Crusafont ICP, Universitat Autònoma de Barcelona, Cerdanyola del Vallès, Spain; 10 Department of Anatomy, University of Helsinki, Helsinki, Finland; 11 Departamento de Ciencias de la Tierra (Paleontología), Universidad de Zaragoza/Instituto Universitario de Investigación en Ciencias Ambientales de Aragón (IUCA), Zaragoza, Spain; 12 Departamento de Prehistoria y Arqueología, Universidad de Sevilla, Sevilla, Spain; 13 Departament de Geologia, Universitat Autònoma de Barcelona, Cerdanyola del Vallès, Spain; 14 ICREA, Barcelona, Spain; 15 Finnish Museum of Natural History, Helsinki, Finland; 16 Visiting Scholars Program, University of Granada, Granada, Spain; 17 Instituto Universitario de la Paz y los Conflictos, Universidad de Granada, Granada, Spain

**Keywords:** Palaeontology, Stratigraphy

## Abstract

Venta Micena is an area containing several palaeontological sites marking the beginning of the Calabrian stage (Early Pleistocene). The richness of the fossil accumulation including species of Asian, African and European origin, makes Venta Micena a key site for the the palaeoecological and palaeoenvironmental study of southern Europe during the Early Pleistocene. Thus, research has been focused on Venta Micena 3, which was originally interpreted as a single palaeosurface associated with a marshy context, in which most of the fauna was accumulated by *Pachycrocuta brevirostris*. Recent excavations have unearthed a new site, Venta Micena 4, located in the same stratigraphic unit (Unit C) and in close proximity to Venta Micena 3. Here we show the first analyses regarding the taphonomic and spatial nature of this new site, defining two stratigraphic boundaries corresponding to two different depositional events. Furthermore, the taphonomic analyses of fossil remains seem to indicate a different accumulative agent than *Pachycrocuta*, thus adding more complexity to the palaeobiological interpretation of the Venta Micena area. These results contribute to the discussion of traditional interpretations made from Venta Micena 3.

The south of the Iberian Peninsula is one of the regions in Europe with the largest number of Early Pleistocene sites. These sites are crucial to the study of the first Early Pleistocene human migrations into Europe. In the Guadix-Baza Basin, the localities of Barranco León (BL) and Fuente Nueva 3 (FN3) have provided clear evidence of human activity dated over ca. 1 million years ago, with the presence of lithic industries^1^, bones presenting cut and percussion marks^2^, as well as human remains^3^. Together with these sites, Venta Micena 3 (VM3) (Fig. 1) is also a well-known, palaeontological locality with no presence of human activity or anthropogenic evidence. Nevertheless, VM3 is strongly characterised by palaeoecological and palaeontological data in which animals of Asian, African and European origin converge^4–9^.

**Figure 1:**
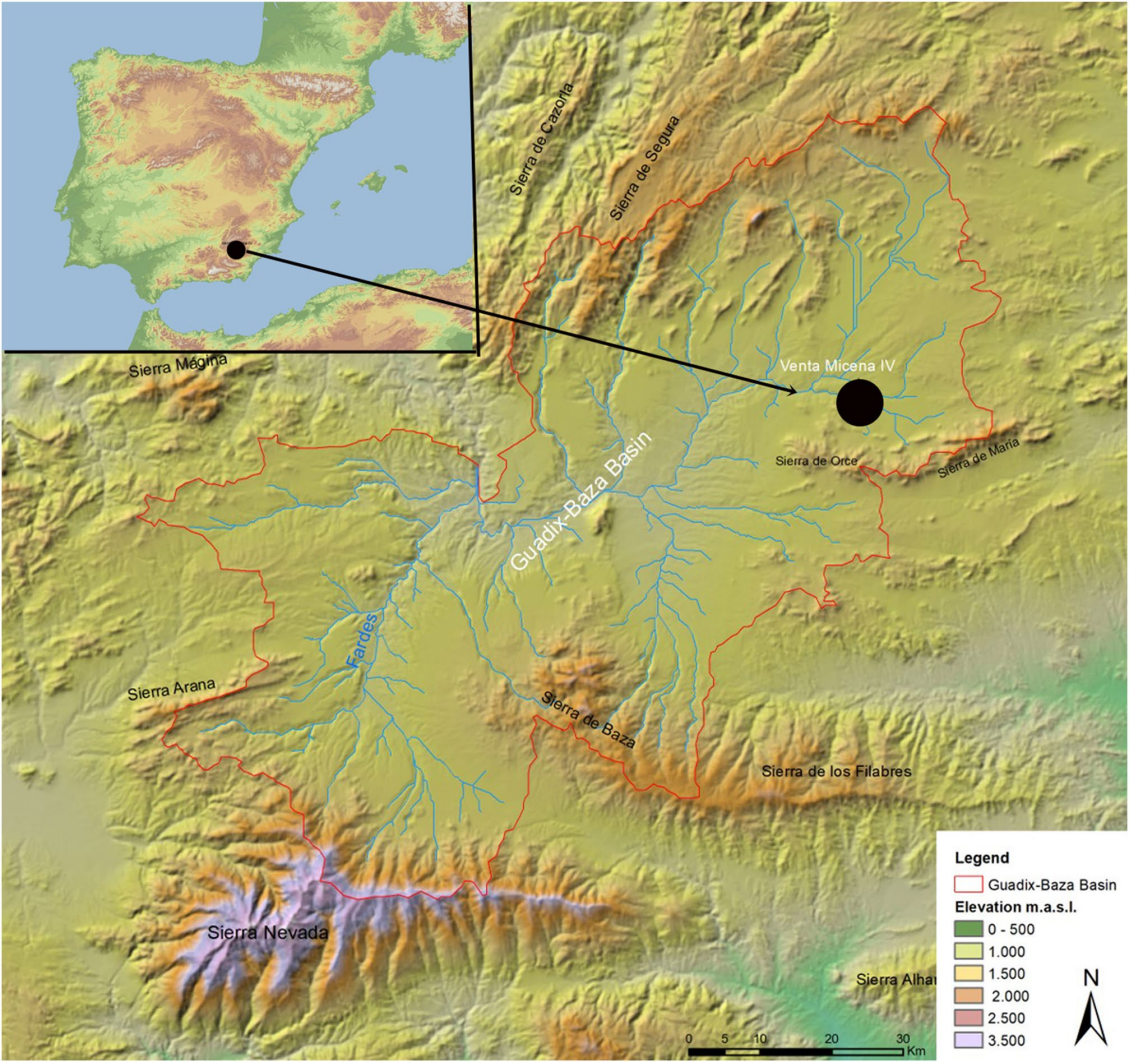
Geographic and topological location of Venta Micena 4.

The great faunal diversity of Venta Micena (VM) sites, and the quality of their remains, have led to the definition of some Pleistocene species for the first time^10,11^. Nevertheless, the VM sites are mainly notable because of their importance in characterizing the palaeolandscape and palaeoclimate of Southern Europe ca. 1.5 million years ago^7^. According to several studies carried out in VM3, the fossil accumulation is currently thought to have been produced by the giant hyaena *Pachycrocuta brevirostris*^12–16^, which makes VM3 one of the primary sites for the study of this super-scavenger’s behavioural attributes^16^. The presence of *P. brevirostris* has been frequently documented throughout Eurasian sites from the Early-Middle Pleistocene, including key sites such as Dmanisi (Georgia)^17^, Vallparadis (Spain)^18^ and Zhoukoudian (China)^19^. In contrast, however, VM3 is devoid of human presence and activity^20,21^, despite the controversial findings reported from the 1980s^22–27^.

The present study describes Venta Micena 4 (VM4), a deposit located in proximity to VM3, and with very similar geological and sedimentological characteristics^28^. Evidence described in VM4, however, has presented contradictory data, namely regarding the stratigraphic and taphonomic data as originally described in VM3, as will be analyzed.

## Geological and chronological context

VM sites are located in the Guadix-Baza Basin, in the southeast of the Iberian Peninsula, forming part of the Baetic Chain (Fig. 1). Located on the northeastern boundary of the Guadix-Baza basin, the Orce region was subjected to the lowstand and highstand dynamics of a large saline lake that dominated the basin. In this sense, it is relevant to point out that while the lake’s shoreline recedes, numerous fresh groundwater ponds would have emerged (Ref.^28^, Supplementary Notes 1, Supplementary Fig. S1). VM has been chronologically framed within the Matuyama magnetic Chron, between Jaramillo and Olduvai. The VM biozone has additionally been classed as MmQ-2, characterized by the occurrence of *Allophaiomys ruffoi* (originally, *A. pliocaenicus*), as well as the absence of suids. These finds have provided an approximate biochronological age of 1.6–1.5 Ma^29^.

VM is located in a white micritic limestone sedimentary environment, having a lateral continuity of more than one kilometer (Supplementary Notes 1). This limestone is part of a sedimentary succession^28,30^ that reveals a landscape made by relatively freshwater shallow lacustrine and palustrine environments (pools and wetlands respectively), not connected with the large saline lake that occupied most of the region [Ref.^28^, Supplementary Notes 1, Supplementary Fig. S1]. The levels where palaeontological remains have been found are located in a unit between 80 to 120 cm thick (Supplementary Notes 1, Supplementary Fig. S2). In general, vertebrate-rich levels are invertebrate-poor, finding the scarce ostracods and gastropods strongly recrystallized due to early carbonate dissolution. VM4 is one of the most prolific sites of the Orce region, currently consisting in a 39 m^2^ excavated window, where abundant fossil remains have been discovered in excavations starting in 2005. The VM sites seem to be slightly older than other archaeological sites in the Orce region, such as BL (1.4 Ma) and FN3 (1.2 Ma)^1,2^. As an example, this is supported by the occurrence of bovid *Soergelia minor,* which is absent in both BL and FN3^31^. On the other hand, the big stenoid horse (*Equus süssenbornensis*), as well as *Ammotragus europeus*, are present in BL and FN3^31^ and are absent. This is consistent with VM presenting an older biochronological age. Finally, VM4 is situated 300 m southwest of VM3 in the same stratigraphical unit (Unit C, Supplementary Fig. S1), having a synchronic in age.

## Results

### Palaeontology

Although the majority of small vertebrates from VM3 and 4 are still under investigation, other VM localities such as VM1 and VM2 (Supplementary Tables 1, Supplementary Tables S1a and S1b), as well as other sites of a similar age in the Guadix-Baza Basin (Cañada de Murcia 1, Fuente Nueva 2, Orce 7), are characterized by a sharp decrease in the diversity of small vertebrate associations when compared with other biozones. Rodent communities are dominated by the vole, *Allophaiomys ruffoi* (89%), a southern and more archaic variant of the Central-European *A. pliocaenicus*. This species is accompanied by representatives of the genus *Apodemus* (*Apodemus sylvaticus* in VM1 and, *A. mystacinus* in VM2^32,33^), *Castillomys rivas*^32–35^ and *Eliomys intermedius* (VM1 and VM2)^32^, *Oryctolagus* cf. *lacosti* (VM1 and VM2) and *Prolagus calpensis* (VM1)^32,36,37^. Furthermore, *Hystrix refosa* (= *H. major*) is also associated with this species in VM2^32^ and *Galemys pyrenaicus* in VM1 and VM2^38^. In VM1, *Galemys* is found associated with *Asoriculus gibberodon*^34^, both of which are indicative of aquatic environments. *A. gibberodon* probably also indicates the presence of patchy landscapes adjacent to water bodies bushes and open lands^39^. The herpetofauna of VM1 and VM2 is represented by *Discoglossus* sp., *Pelophylax* cf. *perezi*, Testudines indet., *Timon* sp., Lacertidae indet. (cf. *Podarcis* sp.), and Ophidia indet^36,40,41^. With the exception of lacertids (*Timon* and *Podarcis*), the remaining taxa documented are indicative of sunny aquatic environments, while *Timon* and *Podarcis* seem to be suggestive of patchy landscapes, open areas and woody zones^34^.

The large mammal associations identified so far in the palaeontological levels of VM4 are comprised of 21 large mammal species, including those belonging to the Felidae, Hyaenidae, Canidae, Ursidae, Elephantidae, Rhinocerotidae, Equidae, Bovidae and Cervidae families (see Supplementary Notes 2). Many of the species recorded at VM3 are also present in VM4 (Table 1, Supplementary Notes 2, Supplementary Table S2), concurring that both sites contain species of African, Asian and European origin^4–9^. Both sites indicate a similar palaeoclimatic setting, dominated by warm and drier conditions than those suggested by the fauna present at BL and FN3^34,42^. The bulk of the fauna is represented by equids alongside *Mammuthus meridionalis*, *Stephanorhinus etruscus* and *Bison* sp. Species from more wooded environments are also frequent, such as two cervids, as well as others from environments close to water sources, such as *Hippopotamus antiquus* (Supplementary Notes 2).

**Table 1:** List of the Venta Micena 4 remains represented by Number of Identifiable Specimens (NISP) and Minimum Number of Individuals (MNI). Abbreviation for the mortality profiles; S: senile, A: adult, J: juvenile, I: infant. %Total represents all macrovertebrates (both carnivores and herbivores). ^a^See in Supplementary Table S3 for caption of species and relation to animal size. See in Supplementary Table S4.

	NISP	% Herbivores	% Total	MNI	% Herbivores	% Total	S/A/J/I	% not adults
*Mammuthus meridionalis*	4	0.3	0.2	2	4.9	3.6	1–0–1–0	50
*Stephanorhinus etruscus*	14	0.9	0.9	5	12.2	8.9	0–1–1–3	80.0
*Equus altidens*	119	8.0	7.4	10	24.4	17.9	1–4–3–2	50.0
*Equus* sp.	5	0.3	0.3	2	4.9	3.6	0–0–1–1	100.0
*Hippopotamus antiquus*	17	1.1	1.1	1	2.4	1.8	0–1–0–0	0.0
*Bison* sp.	43	2.9	2.7	3	7.3	5.4	0–2–1–0	33.3
*Hemibos* aff. *gracilis*	4	0.3	0.2	1	2.4	1.8	0–1–0–0	0.0
*Sorgelia minor*	13	0.9	0.8	1	2.4	1.8	0–1–0–0	0.0
*Capra alba*	19	1.3	1.2	3	7.3	5.4	1–1–1–0	33.3
*Praemegaceros* cf. *verticornis*	61	4.1	3.8	7	17.1	12.5	2–2–2–1	42.9
*Metacervocerus rhenanus*	35	2.3	2.2	6	14.6	10.7	1–3–1–1	33.3
Cervidae indet	1	0.1	0.1					
Herbivore indet. size 0*	3	0.2	0.2					
Herbivore indet. size 1*	1	0.1	0.1					
Herbivore indet. size 2*	71	4.8	4.4					
Herbivore indet. size 3*	96	6.4	6.0					
Herbivore indet. size 3a*	55	3.7	3.4					
Herbivore indet. size 3b*	198	13.3	12.3					
Herbivore indet	733	49.1	45.6					
Total Herbivore	1492	100.0	92.7	41	100.0	73.2		

		% Carnivore			% Carnivore			
*Canis mosbachensis*	15	17.4	0.9	1	9.1	1.8	0–1–0–0	
Canidae	18	20.9	1.1					
*Xenocyon (*= *Lycaon) lycaonoides*	8	9.3	0.5	**2**	18.2	3.6	0–1–0–0	
*Vulpes alopecoides*	1	1.2	0.1	1	9.1	1.8	0–1–0–0	
*Pachycrocuta brevirostris*	15	17.4	0.9	2	18.2	3.6	1–1–0–0	
Felidae	1	1.2	0.1					
*Lynx* sp*.*	3	3.5	0.2	1	9.1	1.8	0–1–0–0	
*Homotherium latidens*	1	1.2	0.1	1	9.1	1.8	0–1–0–0	
*Megantereon cultridens*	1	1.2	0.1	1	9.1	1.8	0–1–0–0	
*Panthera* cf*. gombaszoegensis*	1	1.2	0.1	1	9.1	1.8	0–1–0–0	
*Ursus etruscus*	11	12.8	0.7	1	9.1	1.8	0–1–0–0	
Carnivora	11	12.8	0.9					
Total carnivore	86	100.0	5.3	11	100.0	19.6		
Lagomorpha	8		0.5	2		3.6	0–2–0–0	
Testudines	14		0.9	1		1.8	0–1–0–0	
Aves	9		0.6	1		1.8	0–1–0–0	
Total	1609		100.0	56		100.0		

Similarly to VM3, VM4 provides a large number of carnivore remains, including; hyenids (*P. brevirostris*), felids (*Homotherium latidens*, *Megantereon cultridens*, *Panthera* cf. *gombaszoegensis*, *Lynx* cf. *pardinus*) and canids (*Xenocyon* (= *Lycaon) lycaonoides*, *Canis mosbachensis*, *Vulpes alopecoides*) (Supplementary Notes 2).

### Taphonomy

The bulk of fauna from VM4 is represented by herbivores, comprising over 90% of the fossil record. *E. altidens* is the most abundant taxon, both in frequency of remains and number of individuals, contributing to 24.4% of identified herbivores and 18.5% of all individuals at the site (Table 1). Cervids are also an important component of the assemblage, followed by bison, caprines and megaherbivores, such as elephants, rhinoceroses and hippopotamuses (Table 1). *Pachycrocuta* are the most representative carnivore in the assemblage, followed by large felids, canids (wild dog-like canids, foxes and wolves), bears, and finally smaller felids such as lynx (Table 1).

Mortality patterns reveal that individuals of all ages are recorded in VM4, with a relatively higher amount of non-adult individuals. Among the large-sized species present, the number of non-adults among elephants and rhinoceroses is similar to, or higher than, that of adults (Table 1). Among medium-sized species, *E. altidens, Hemibos* aff. *gracilis* and *Praemegaceros* cf. *verticornis* are represented by similar amounts of adults and non-adults. The only exception to this is in the case of bison, where non-adults only represent 33% of the individuals (Table 1). Finally, among smaller species such as caprids and small cervids, adults outnumber new-born and juvenile individuals (Table 1). Nevertheless, the total number of individuals in each species is too low to draw reliable conclusions on the resulting patterns. From this perspective, a prime-dominant, L or U shaped mortality profile cannot be clearly discerned.

Regarding skeletal profiles, teeth are by far the most abundant anatomical elements, comprising of 36.9% of the faunal remains (Supplementary Tables S4, S4a). Species of size classes 1 (25–50 kg) and 5 (> 1000 kg), such as elephants, rhinoceroses and hippopotamuses, are not representative due to the scare number of remains (Table 1 and Supplementary Tables S4, S4a). This is also the case for carnivores.

Species of size class 2 (50–125 kg), such as *M. rhenanus, C. alba or S. minor,* show biased skeletal profiles, with a predominance of teeth (55% of the sample), as well as anterior limbs (scapulae, humerii, radii, carpal bones and metacarpals). This is almost twice the number of posterior limbs (pelves, femora, tibiae, patellae, tarsal bones and metatarsals) (Supplementary Tables S4, S4a).

Sizes 3, 3a and 3b species (125–500 kg), on the other hand, are well represented by all anatomical elements. Cranial elements, together with teeth and mandibles, predominate, accounting for 30% of determinable bones, while axial and appendicular elements are also well represented with frequencies both higher than 20% (Supplementary Tables S4, S4b). Nevertheless, although all skeletal regions are represented, in some cases a certain bias is observed. An example of this can be found in the disproportionate amount of posterior limb remains, as well as a modest number of anterior limb specimens (Supplementary Tables S4, S4b), which contrasts with the more balanced representation of these elements observed in VM3^7^.

The VM4 fossil remains show a moderate fragmentation. Only 36% of remains measure less than 3 cm (Table 2), with more than 55% of long bones presenting green fractures (Table 2). In addition, some bones have been documented in anatomical connection. Examples include the attached humerus-radius of *S. etruscus* (NE area of the site), as well as a femur-tibiae, fibula and talus of this same species (SW area of the site). Also, a set of eight cervical vertebrae of *M. meridionalis* was retrieved on the western edge of the site. The almost complete forelimb of a *X. lycaonoides* individual was also found in the centre of the site, as well as two complete hindlimbs of the same species (NW corner of the site). Finally, two hemipelves belonging to an *E. altidens* individual was found towards the west*,* all of which present a good representation of bones found in anatomical connection at VM4.

**Table 2:** Taphonomical characteristics of Venta Micena 4.

Taphonomic characteristics		NISP	%	%Representation
	Total amount of specimens	1609		
	Total amount of specimens excluding teeth	1374		
Fragmentation	Bones < 3 cm	585	36.4	
	Bones 3.1–5 cm	382	23.7	
	Bones > 5.1–9.9 cm	382	23.7	
	Bones > 10 cm	260	16.2	
	Long bone with green fracture	186	55.1	
	Long bone with dry fracture	152	44.9	
Bone surfaces	Badly preserved specimens	427	31.1	% respect to total amount of specimens excluding teeth
Weathering	Weathering stage 1–2	148	9.2	% respect to total amount of specimens
Water alteration	Abrasion	642	39.9	% respect to total amount of specimens
	Light stage abrasion	189	29.4	
	Intermediate stage abrasion	242	37.7	
	Intense stage abrasion	211	32.9	
	Calcitic concretions	155	9.6	
	Oxide staining	307	19.1	% respect to total amount of specimens
	Biochemical alterations	402	25.0	% respect to total amount of specimens
Carnivore activity	Bones with tooth marks	43	4.5	% Excluding bones with badly preserved bones and teeth
	Bones with tooth Marks with pits only	38	88.4	
	Bones with tooth marks with scores only	7	16.3	
	Bones with tooth marks with both pits and scores	4	9.3	
	Bones with punctures only	1	2.3	
	Bones with furrowing	17	4.5	
	Long bones with furrowing	9	5.8	% with respect to long bones
	Rodent tooth marks	1		

Bone surfaces are also well preserved, with only 31% of the remains presenting poor preservation (Table 2). Such a high preservation rate can be the result of several factors, such as the low occurrence of weathering. From this perspective, only 9% of faunal remains have been observed to reach weathering stages 1–2^43^, indicating short or/and low subaerial bone exposure. Diagenetic alterations are also rare, and are often limited to manganese oxide stains and calcite concretions (Table 2). Evidence of hydric alterations are limited to abrasion (which affects a 40% of specimens), without the presence of rounded bones. Nevertheless, only 33% of these specimens show an intense degree of abrasion, implying hydric alterations to be notably low. On the other hand, 25% of the remains show alterations of biological origin, including biochemical corrosion as well as root-marks. Nevertheless, in most cases the impact of these alterations is low to moderate.

Finally, carnivore alterations were only observed on 4.5% of the well-preserved bones (Table 2). Only 3 bones show 3–6 tooth marks, while the remainder of tooth marks bones present no more than 2. Furthermore, salivary and gastric alterations are absent, with a similar lack of coprolites. Regarding tooth mark typologies, pits predominate over scores, accounting for 88% of the documented tooth marks (Table 2). Most of the tooth marks are distributed on long bone diaphyses and axial elements (Table 3).

**Table 3:** Bones with tooth marks. Epd: Distal Epiphyses. Shaft: Diaphyses.

Bones with tooth marks	Species size class					
	2	3	3a	3b	5	Indet
Vertebrae				1		
Scapulae	1					
Humerii	1 shaft			5 shaft	1 shaft	
Radii			1 epd	1		
Metacarpals				2 shaft 1 epd		
Pelves				1		
Femora				2 shaft		
Tibiae				6 shaft 1 epd		
Metatarsals	2 shaft					
Calcanei	1					
Long bone shafts		1	1	8		1
Indeterminate			1	3		1
NR total tooth marks	5	1	3	31	1	2

Extensive bone deletion is not frequent, but it has also been observed, with only 4.5% of the tooth-marked bones presenting evidence of furrowing (Table 2). Likewise, several taphotypes (1, 3, 4, 5, 6, 11 and 15), proposed by Ref.^44^, have been documented on long bones with crushed epiphyses (Table 4). Numerous long bones additionally present evidence of green fractures (Table 2), with the additional presence of different notches types (Table 5). Finally, complete long bones are rare, while bones that preserve > 50% of the total shaft circumference are also scarce (Table 6).

**Table 4:** Taphotypes observed on different long bones according to (44).

Taphotype No.	Skeletal element	NR total	%
0	Tibia	2	10.5
1	Humerus	1	5.3
3	Tibia	5	26.3
4	Humerus	1	5.3
5	Tibia	1	5.3
6	1 Radius, 1 Tibia	2	10.5
11	Radii	1	5.3
15	1 Femur/3 Humerii/1 Radius/1 Tibia	6	31.6

**Table 5:** Evidences of notches observed in VM4.

Notches	NISP	%
Single	8	14.3
Opposing	3	5.4
Incomplete type A	11	19.6
Incomplete type B	2	3.6
Incomplete type C	3	5.4
Double	3	5.4
Double opposing	4	7.1
Pseudonotch	1	1.8
Micronotch	15	26.8
Multiple	6	10.7
Total	56	100

**Table 6:** Degree of total circumference and fragment length vs complete bone length. Only green fractured bones are included.

Degree of circumference	NISP	%	% Length respect to the total length of the bone	%
< 25%	126	67.75	136	73.1
25–50%	33	17.75	40	21.5
> 50%	27	14.5	10	5.4
Total	186	100.0	186	100.0

### Spatial analyses

Artificially intelligent systems for the identification of discrete fossiliferous levels revealed 2 distinct and independent bone concentrations levels that could be clearly identified across the entire 39 m^2^ extension of the VM4 site. These levels have been subsequently named Level I (VM4-I) and Level II (VM4-II). VM4-II is located directly above VM4-I, approximately 200–230 cm below the surface with a relatively homogeneous horizontal spread and slight NE-SW dip. VM4-I, on the other hand, is located approximately 250–280 cm below the surface, and is observed to be a much denser horizontal plane.

Each bone accumulation has a local thickness of generally < 30 cm. VM4-I and VM4-II are vertically scattered 50 and 30 cm (i.e., stratigraphic heights between 0 and 50 cm and between 60 and 90 cm, respectively). The separation between VM4-I and VM4-II is defined by a 10 cm interval.

**Figure 2:**
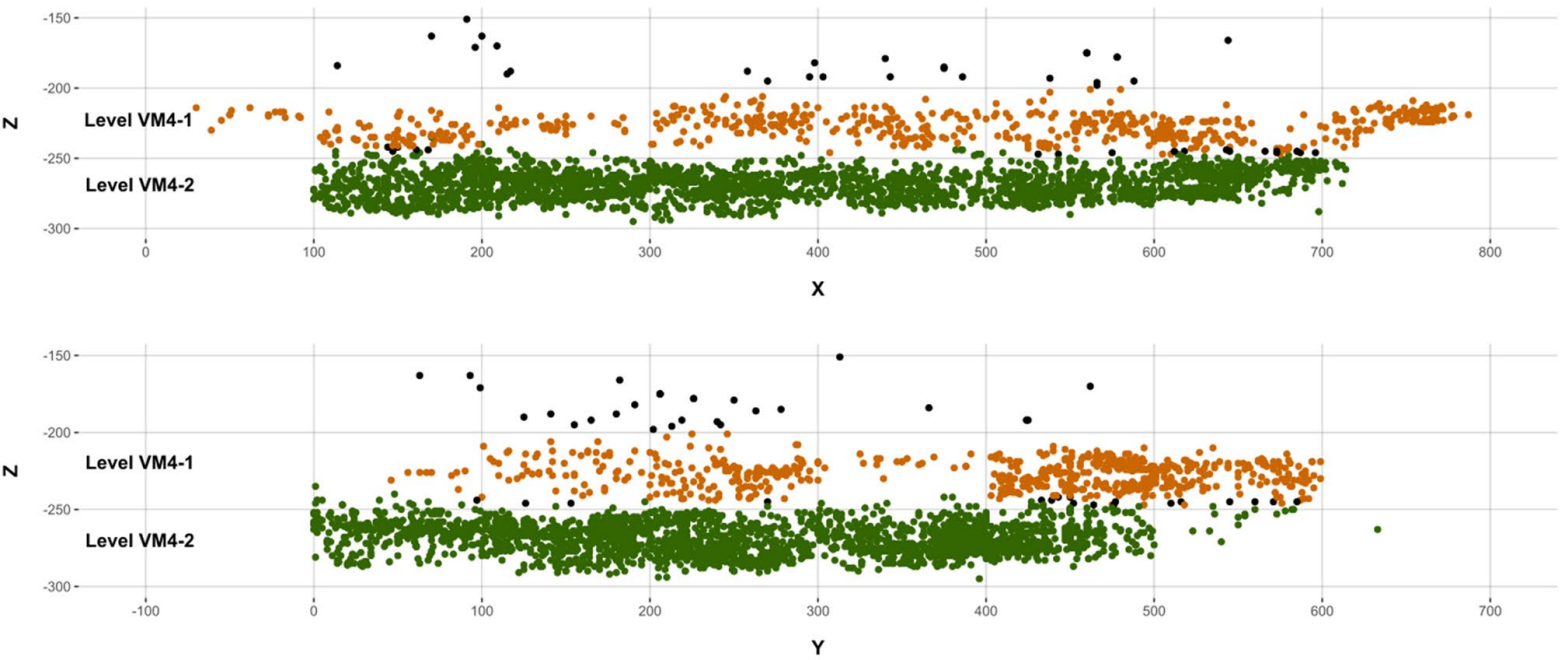
Scatter plot showing the spatial distribution of levels VM4-I (dark green) and VM4-II (brown), as identified using artificially intelligent systems. Black points indicate indeterminable points with < 80% confidence when being assigned to a level.

When evaluating the quality of these defined levels, Random Forest (RF) algorithms proved to be the most confident models when associating each of the finds to their corresponding fossiliferous levels. RF, on average, presents a confidence of 100 [+ 0.0, − 0.0]% probability when making new predictions. Support Vector Machines (SVM), on the other hand, saw a slight drop in confidence, assigning most finds a class probability of 99.9 [+ 0.001, − 0.003]%. When considering the performance of both models in a system, both algorithms were successful in assigning 4219 fossils to a particular level; 3482 fossils were assigned to Level I, 737 to Level II, and 76 remains were considered indeterminable with < 80% confidence when assigning finds to any particular level. Among the classified remains, both SVM and RF agreed on the allocation of 97.5% of these remains. When disagreement did occur, RF appeared to be the most decisive algorithm at least 87.1% of the time. Detailed evaluation of agreement-disagreement rates additionally reveals an inter-rater reliability of 0.85, with near perfect agreement according to Cohen’s κ. Under this premise, while RF is in general a more confident classifier, the use of both algorithms in combination provides a more robust overall classification of the entire site (Fig. 2).

**Figure 3:**
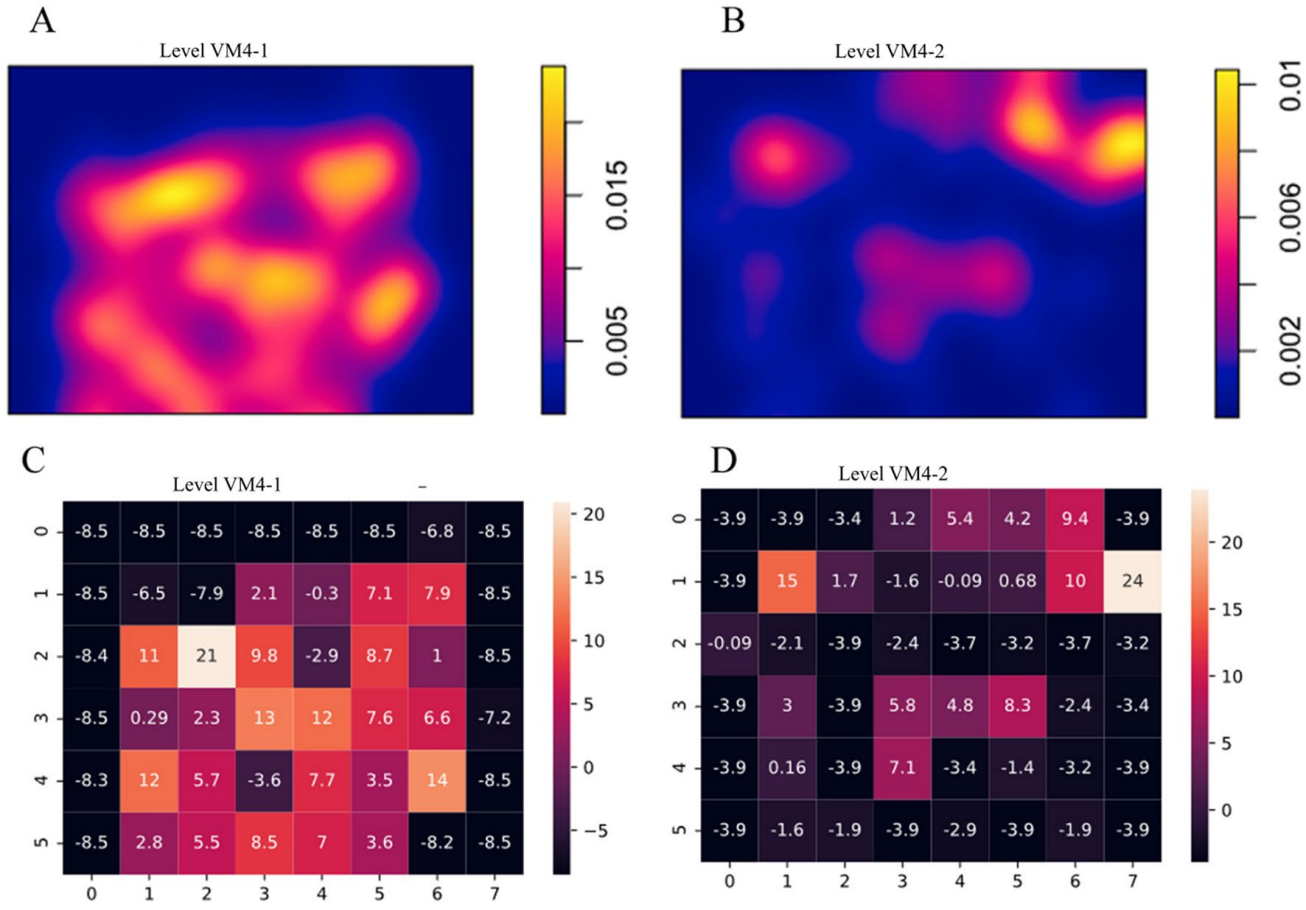
Density (**A**, **B**) and heat (**C**, **D**) maps for Pearson residual counts (for each of the level VM4-I (**A**, **C**) and VM4-II (**B**, D).

**Figure 4:**
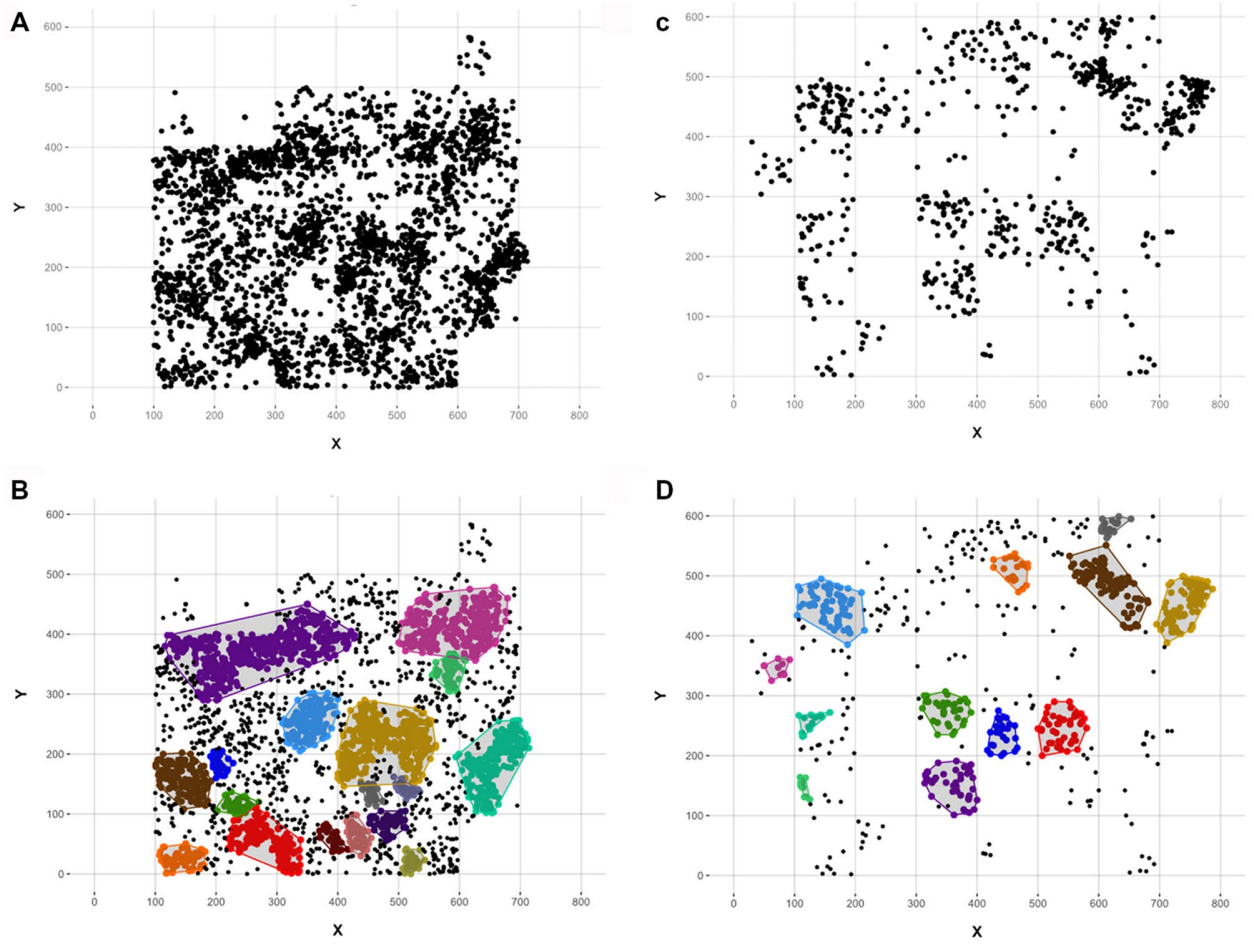
Empirical (black) and theoretical (red) spatial correlation functions for Venta Micena 4 levels I and II using Ripley’s *K* and Besag’s *L*. Empirical functions account for border correction estimates while both for *K* and *L* the inhomogeneous variants of these tests were performed.

Detailed statistical analyses of each of these levels reveal VM4-I to present a strong concentration of faunal remains (Fig. 3a), with the highest accumulated density of fossil remains per m^2^. Similarly, VM4-II shows higher concentrations of remains, which are oriented towards the NE (Fig. 3b), while VM4-I is slightly more spread out. Overall, Monte Carlo tests for Complete Spatial Randomness (CSR) reveal inhomogeneous distribution patterns for both VM4-I (χ^2^ = 3297, *p* = 2e−04) and VM4-II (χ^2^ = 1582, *p* = 2e−04), while few quadrats comply with CSR (Fig. 4c,d).

Upon analysing spatial correlations with theoretical *K*(*r*) functions of an inhomogeneous Poisson process, both VM4-I and VM4-II can be seen to present general tendencies for more cluster-like patterns, as confirmed by the centered *L*(*r*) function (Fig. 4)*.* While VM4-II shows slight tendencies towards a regular point process, this is likely due to the smaller sample size and lower concentrations across the overall surface area. It is worth noting that Hopkins–Skellam tests are able to confirm that both levels present strong tendencies towards clustering across the overall spread of the spatial window (VM4-I: A = 0.01, *p* < 2.2e−16; VM4-II: A = 0.06, *p* < 2.2e−16).

**Figure 5:**
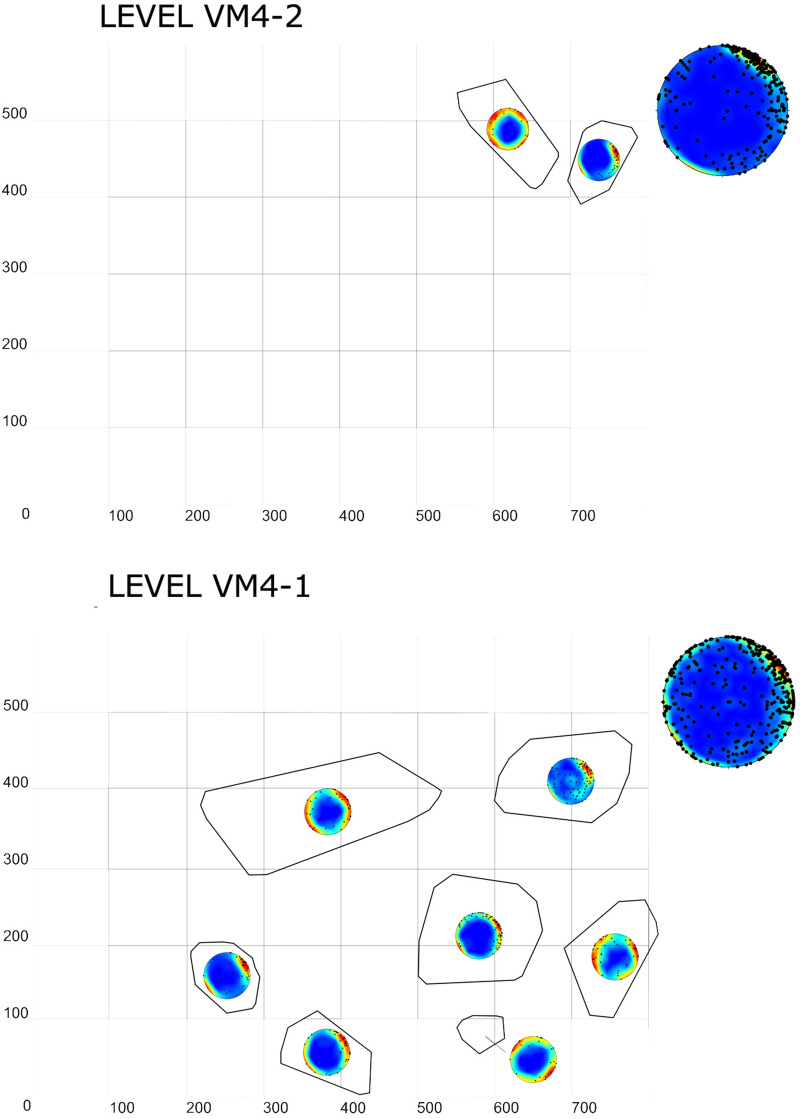
Spatial distribution of fossils recovered from both VM4-I (**A**, **B**) and VM4-II (**C**, **D**). (**A**, **C**) Raw spatial coordinates of fossil finds. (**B**, **D**) Clusters calculated using Density Based pattern recognition algorithms.

Upon quantifying the location of clusters through density based pattern recognition algorithms, 17 clusters were detected in VM4-I and 12 clusters in VM4-II (Fig. 5).

**Figure 6:**
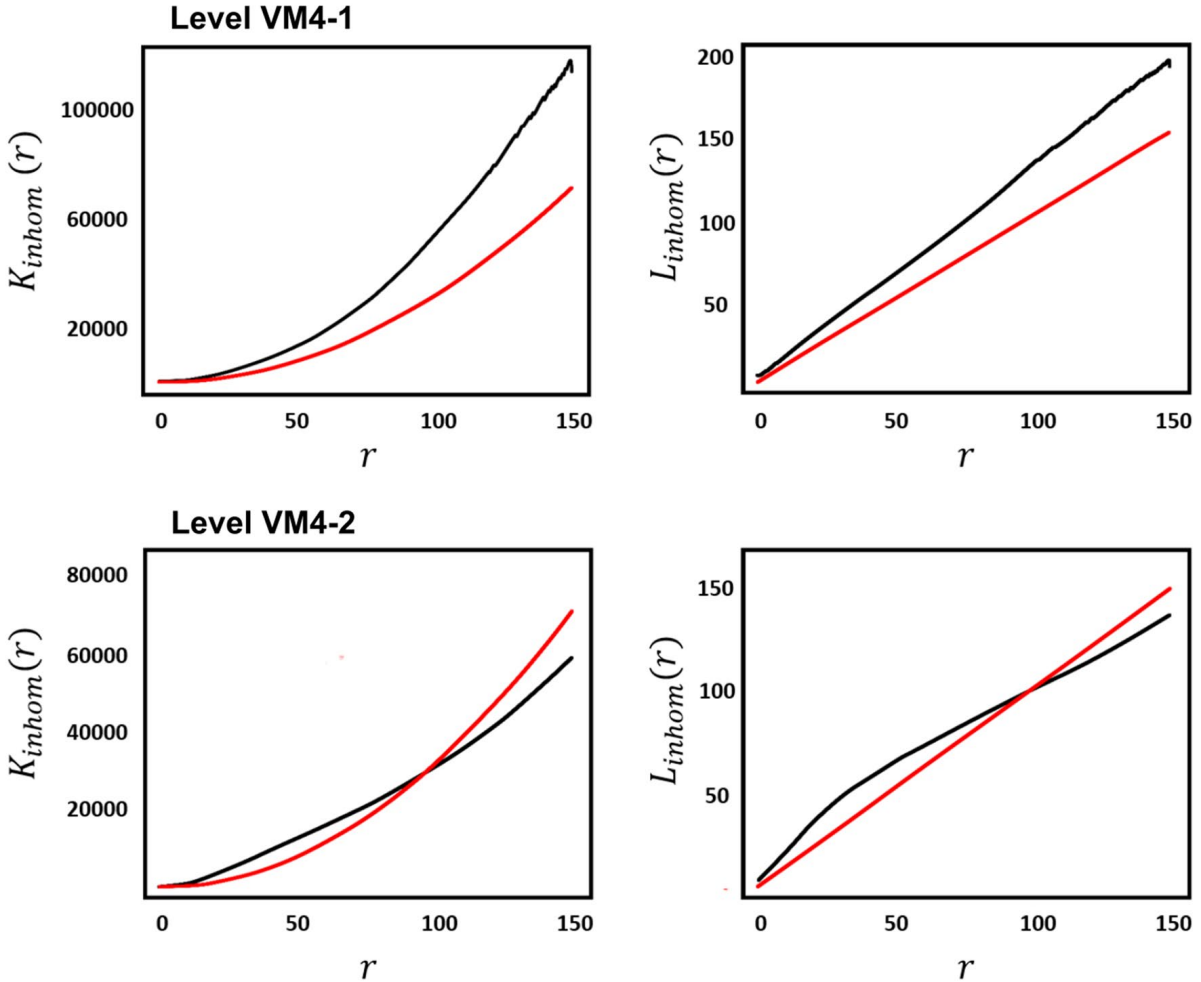
Stereograms presenting general orientation and plunge patterns across both the VM4-I and VM4-II levels. Localized stereograms were obtained according to the detected clusters in Figure. Numeric data relating to these graphs can be consulted in Supplementary Methods.

When considering orientation patterns for fossil remains, only 1396 fossils were documented with orientation values (VM4-I: n = 1125; VM4-II: = 271). Nevertheless, in both levels preferential orientations towards the NE (VM4-I = 34.65°; VM4-II = 45.33°) have been documented and calculated to be of notable importance (see Supplementary Methods). When analysing orientation patterns across the site (Fig. 6), general trends reveal most clusters to share a similar central tendency, with most clusters being oriented between the NNE and the ENE. Two clusters in VM4-II show exceptions to this rule with a slight tendency towards the NW and NNW, however this only represents 8% of the total sample for this level. Similarly, with the exception of 3 clusters (2 in VM4-I: 34% of the sample; 1 in VM4-II: 36%), preferential orientations are strong across the entire site (See orientation uniformity data from Supplementary Methods, Supplementary Table S5).

With regard to the general slope of fossil finds, only 4% present extreme azimuth values over 45°, while 79% of finds have been recorded relatively flat along the surface (Fig. 6).

**Figure 7:**
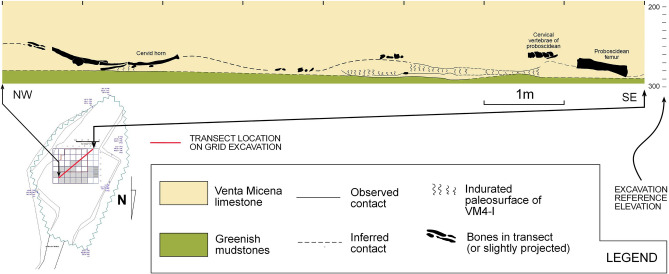
Topographic section of VM4.

When combining information, VM4 in general therefore presents a strong tendency for relatively flat slopes and NE–SW orientations, likely conditioned by the natural topography of each palaeosurface (Fig. 7). Needless to say, when considering the natural topography of each level, gravity is the likely cause for the observed patterns at VM4 (see Supplementary Methods, Supplementary Table S5).

## Discussion

The present study of VM4 reports evidence suggesting this site to be a palimpsest of various events, distributed over several palaeosurfaces^28^. This is corroborated by the vertical and spatial distribution of faunal remains throughout the stratigraphic sequence (Figs. 2, 3, 4, 5).

Taphonomic information has additionally provided an insight into the rate at which fossils were buried, revealing VM4 to be in the context of a series of short-time events, followed by rapid sedimentation. This is supported by the low degree of weathering (Table 2, in accordance with Ref.^43^), low tooth mark frequencies, and also by the presence of some remains found in anatomical connection. From a different perspective, the absence of rounded bone surfaces helps confirm fluvial currents to not be responsible for the accumulations present at VM4 (Table 2). Nevertheless, while hydraulic activities did not move the remains, sedimentary abrasion has been observed to have affect bone surfaces, product of circulating water moving mobile sediments over the bones. While these currents were not strong enough to remove osteological remains, sedimentary abrasion has had an impact on bone surface preservations, resulting in the poor preservation rates observed across 31.1% of specimens.

**Figure 8:**
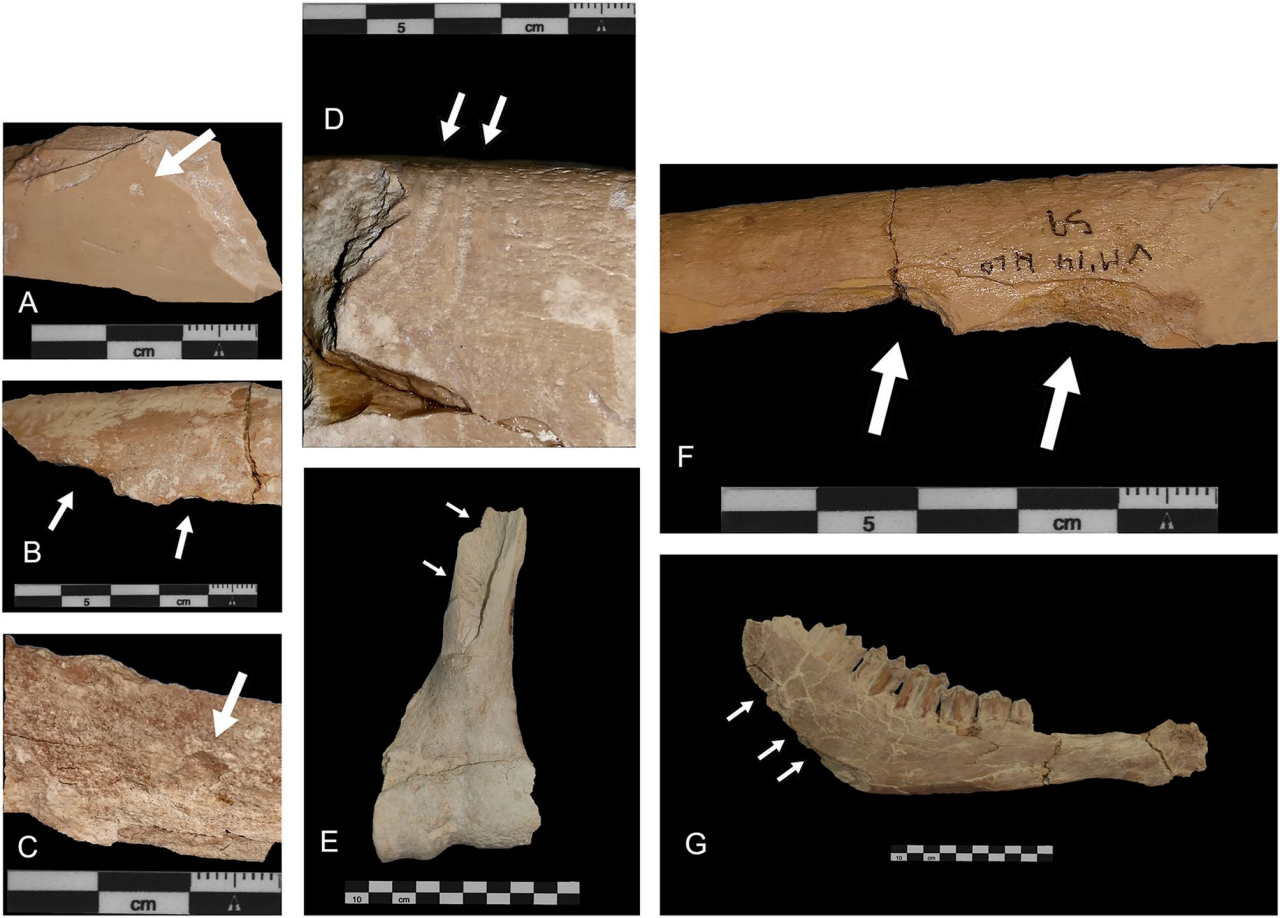
(**A**) A single pit on a long bone fragment; (**B**) Pseudo-notches on a long bone fragment; (**C**) a single pit on an non identifiable bone splinter; (**D**) Two scores on a long bone diaphysis; (**E**) Evidences of proximo-distal consumption of an *E. altidens* right humerus, with furrowing marks in the medial diaphysis; (**F**) Double notch on a long bone fragment; (**G**) Furrowing on the jaw angle and ramus of a *Bison* sp. mandible.

As seen by the presence of tooth marks (Fig. 8), furrowing (Table 2), and notches (Table 5), the influence carnivores had in the formation of VM4 is undeniable. Although insufficient information is currently available to discern the precise carnivore agencies present at this site, some important conclusions can be drawn about the activity of carnivores and their role in the formation of the fossil assemblage.

In general, while the impact carnivores had on VM4 is notable, carnivore activity in general can be considered of a low intensity. This can be seen by low tooth mark frequencies, the reduced number of bite marked bones (4.5%, Table 2), the low number of tooth marks per bone (< 2 marks per bone, Table 2), the absence of digested bones and salivary alterations, the absence of coprolites, and the moderate percentages of bones with furrowing (4.5%, Table 2). These observations contrast with the taphonomic data present at other *P. brevirostris* bone assemblages, such as Vallparadís^45^, Zhoukoudian^19^, VM3^12,13^ and Fonelas P-1^46^. The same can be said of other extant carnivore accumulations in South African sites^47^, among others^48–51^. In light of this, it can be concluded that VM4 should not be considered a den site, nor product of *Pachycrocuta* activities, as described in the case of VM3^7,12–15^.

The taphonomic evidence described in VM4 also differs greatly from bone accumulations typically associated with carnivore dens or open air rendezvous sites. Bone accumulations from hyaena dens, for example, tend to be characterized by high frequencies of tooth marked bones, as well as a high frequencies of tooth marks per specimen. Similarly, other diagnostic criteria for detecting intensive hyaena activities include; gastric alterations, high amounts of furrowing, absence of epiphyses, abundance of bone cylinders, as well as remains of infant carnivores^47,49,52,53^. This profile does not fit the case of VM4, considering the low tooth mark frequencies, the low number of tooth marks per specimen, as well as the absence of coprolites, digested bone, and general salivary alterations (Table 2).

When compared with the accumulations produced by felids, both leopards^50,54^ and lions^51^ usually leave complete skeletal profiles, while elements of the axial skeleton are often well represented. Felids are also known to leave carcasses in anatomical connection, leaving most bones complete^47,50,51,54^, presenting low tooth mark frequencies^51^, similar to the frequencies described here in VM4. Nevertheless, VM4 differs from felid assemblages as seen with skeletal profiles not dominated by axial bones, while most faunal remains are not found in anatomical connection. Similarly, the VM4 assemblage presents numerous specimens with notches (Table 5), a feature uncharacteristic of felid activities, while taphotype number 15 (bone cylinders) is also present. Finally, pits clearly predominate over scores, another feature uncharacteristic of felids (Table 2).

Beyond these comparisons, the VM4 assemblage is also characterized by the presence of all skeletal elements, with a slight predominance of teeth (Supplementary Tables S4, S4a, S4b). Similarly, bone modifications characteristic of carnivores are abundant, including tooth marks (Fig. 8), notches (Fig. 8), taphotypes and green fracture planes. All the aforementioned evidence can be found in kill sites, where some carnivores hunt their prey, others scavenge, and others disperse the remains. To this effect, VM4 would have been a place used recurrently by different carnivores to hunt, presenting primary access to their prey. This scenario would, throughout time, produce a palimpsest comprised of periodic hunting events in the same space. In addition although coprolites are absent from VM4, this may be due to the fact that feces are no always present in recent kill sites^55,56^.

Despite the low impact of carnivore activity, the activities of carnivores could have caused a bias on several skeletal portions, decreasing the frequency of complete long bones in the site (Table 6, Supplementary Tables S4a, S4b). The scarcity or absence of some anatomical regions could be product of the dispersion or transportation of the carcasses made by the predators when obtaining the prey, and by the consumption that the carnivores would carry out after hunting the prey.

This skeletal bias is observable in Size 2 animals (Supplementary Tables S4a, S4b), which are represented mainly by teeth, while other skeletal elements are very poorly represented. As abiotic agents are not relevant in this accumulation, and in general terms bone preservation is good for small and medium sized animals, such bias could be due to the greater ease of transporting a smaller carcass than a larger one, thereby increasing the bias of smaller animals. Only most of the skeletal elements of Size 3 species are preserved, although bias is still present as seen through the lower representation of anterior quarters (Supplementary Tables S4a, S4b).

Regarding the carnivore that may have intervened in VM4, evidence seems to suggest the important activity of hyaenas or canids. This can be derived from pit dominance, presence of bone cylinders, presence of notches, furrowing, epiphysal collapse, and a scarcity of axial bones. Nevertheless, evidence also seems to suggest some felid activity in the area, considering the low tooth mark frequencies and small number of tooth marks per bone. This situation presents some complications, especially when considering the poor documentation available for other carnivores in the VM area, including *P.* cf. *gombaszoegensis* or *X. lycaonoides*. The taphonomy of the jaguar suggests that they have a greater impact on bones when they first access a carcass as opposed to the case of other felids^57^, normally collapsing the epiphyses. Painted dogs (e.g. the genus *Lycaon*), on the other hand, show lower tooth mark frequencies than wolves, while producing fewer tooth marks per bone and not much furrowing^58^.

According to these observations, the carnivore activity at VM4 could have been induced by various carnivores, whose precise actions will be topic of future investigations including larger palaeontological samples and aided by new methodologies^59–61^. When considering the geological characteristics described in Ref.^28^, this site can be interpreted as a locality close to where herbivores would recurrently have access to drinking water, and are thus easy prey for carnivores. This is a frequent phenomenon observed in typical waterholes of the African savannah.

Mortality patterns of VM4 are characterized by the concurrence of infantile individuals for all herbivore species, with the exception of *S. minor*, *H*. aff. *gracilis* and *H*. *antiquus* (Table 1). Young individuals (Juvenile and Infants) are also represented in the VM4 fossil record, especially in the cases of *E. altidens, S. etruscus* and *P.* cf. *verticornis.* In addition, senile individuals are present, as are the cases of *P*. cf. *verticornis*, *M*. *rhenanus*, *C*. *alba* and *E. altidens* (Table 1). Finally, age patterns are completed with the appearance of adult or prime adult individuals, although they never present frequencies higher than the combined sum of infantile-juvenile and senile (Table 1). This mortality pattern is consistent with those observed by Refs.^55,62,63^, but slightly different than the ones from Refs.^64–68^, thus implying that these profiles be more similar to those produced by the hunting patterns of carnivores. Nevertheless, future studies should look into the mortality patterns of VM4, especially with those typical of kill sites in comparable landscapes. These could include, natural and seasonal ponds, or the margins of relatively shallow lacustrine and palustrine water body environments.

From a different perspective, it is also important to consider the similarities and differences VM4 has with the observations and interpretations made at VM3.

Firstly, similar species are present at both sites, including *S. etruscus*, misidentified in previous publications as *S. hundsheimensis*^9,29,69^. As for the rest of taxa, both sites show a dominance of species from open environments, with a few taxa more typical of wooded as well as aquatic environments.

Secondly, and from a taphonomic perspective, fluvial activity has not played a significant role in the formation of this site, as implied by the low degree of weathering and the spatial distribution of specimens from both sites. A rapid burial of the remains is also proposed, given the low incidence of biochemical alterations on the fossil remains^8,12–14,70^. In fact, only 10.7% of the remains show weathering stages 1–2 in VM3^12,70^, similar to the frequencies of those described in Table 3 for VM4. Only 5% of the VM3 specimens show biochemical alterations, while 25% exhibit this kind of modification in VM4. Nevertheless, these percentages are based on a relatively small sample size.

Concerning fluvial alterations, both sites show scarce evidence of water activity, as has been documented in the present study (Table 2), as well as in VM3^12,70^. The absence of taphonomic features related with these types of environments indicates that fluvial activity was not a significant factor in the formation of both sites. From a similar perspective, spatial data does not present patterns associated with flowing water^71–73^.

While stereoplots and orientation data from VM3^69,74^ reveal randomly distributed remains, with no preferential orientations^7,12,69,75,76^, spatial data of VM4 points to preferential orientations (Figs. 5, 6, Supplementary Table S5). Nevertheless, the overall taphonomic characteristics indicate a certain incompatibility with fluvial action. Alternatively, terrain irregularities (Fig. 7) would indicate that palaeotopography would play an important role in the accumulation. Nevertheless, it is worth to note that the abrasion present in the bones indicates that hydraulic activity was important, not in the generation of the accumulation but in its reconfiguration. In other words, the bones are oriented as they would adapt to the previous lineaments of the palaeosurfaces.

In the same way as in VM3, anatomical connections have also been described, reinforcing the great similarity between both sites. Nevertheless, an important difference between VM3 and VM4 is that the former was studied as a single bone accumulation, while the later includes at least, two different fossiliferous levels. While this has not been carried here, it will be important to characterise the taphonomic patterns involved in both levels in future analyses.

A second important difference is that, while the accumulative agent in VM3 was originally described as being *P. brevirostris*^7–9,12–15^*,* in VM4 we do not know the taphonomic agent responsible for the formation of the site. If both sites share the same chronology, the same palaeofaunal diversity, and similar palaeoenvironmental and palaeoclimatological implications^28^, the findings from VM4 could suggest that VM3 may also present multiple discrete fossiliferous levels, which will therefore require a different approach to defining the taphonomic history of this iconic site.

Although we cannot specify which carnivores were involved in the fossil accumulations of VM4, the taphonomic profiles described suggest that *P. brevirostris* was not the main accumulating agent. The patterns of tooth marks described, the distribution of tooth marks, the number of tooth marks per bone, and the relatively low index of bones with furrowing, differ greatly from those described in the accumulations produced by *P. brevirostris*^7,12–19,69,76^. This provides an interesting point of debate for the interpretation of both VM3 and VM4, adding to their complexity.

## Conclusions

According to the evidence described in the present study, VM4 is a palaeontological site of similar age and with similar characteristics to VM3. Both are characterized by mammalian assemblages dominated by equids, typical of open, shrubby landscapes. Similarly, the location of VM3 and VM4 on the margin of relatively shallow lacustrine and palustrine environment makes them a favourable habitat for hippopotamuses, an animal found in both sites.

From a taphonomic perspective, VM4 has been interpreted as a bone assemblage formed at the margins of a freshwater body, an environment ideal for the hunting grounds of carnivores and their lingering prey. This contrasts with the interpretations of the nearby VM3 site, interpreted as a *P. brevirostris* den. The identification of two fossiliferous levels in VM4 indicates a multi-event depositional scenario, an observation that also contrasts with the single formational event proposed for VM3.

Likewise, the material from VM4 has allowed us to revise and redefine the VM faunal list, which has been significantly updated with regards to previous versions (especially for some particular groups such as Rhinocerotidae).

Nevertheless, the definition of these two new palaeostratigraphic levels, as well as the mortality patterns and skeletal bias presented in this paper, raises interesting questions about the relationship between VM3 and VM4 that still remain unanswered.

To date, research is still underway at VM4. Further work will therefore attempt at discerning the precise carnivores involved in the formation of this site, identifying the presence of a single or multiple predator types. Future investigation will also make an effort at characterising the two separate depositional events discovered in the present study. It will be of great interest to know what implications this has for the interpretations of VM3.

## Methods and sample

The bone sample analysed at VM4 comprises of 1609 remains (Table 1), distributed over a surface area of 39 m^2^, and recovered from the 2005, 2018 and 2019 excavation field seasons. This sample has been analysed from a palaeontological and taphonomic perspective. Together with these remains, spatial and stratigraphic information on the coordinated fossils from the 2005, 2014, 2015, and 2017–2019 excavations have been included.

### Large mammals palaeontology

Taxonomic identifications were based mainly on teeth and diagnostic bones. Available reference materials from the provincial Museum of Granada were also used, as well as general and species specific bibliography (Refs.^77,78^, Supplementary Notes S2). With regard to the palaeoecological and palaeoenvironmental implications for the represented taxa, herbivorous species were divided into the following three groups; woodland dweller, open-land species and water sources.

### Taphonomy

Many specimens were identified both anatomically and taxonomically, while there are numerous others that were only identified anatomically. These specimens were assigned to weight/size classes using comparative bone data of both carnivores and herbivores. Herbivores were assigned to 5 different size classes; Very Small size (0) for species less than 25 kg of weight; Small Size (1), including species weighing 25–50 kg; (2), including species weighing 50–125 kg; Intermediate size (3), including species weighing 125–500 kg, with a subdivision of 3a (125–250 kg) and 3b (250–500 kg); (4), including species weighing 500–1000 kg; and very large species (5), weighing > 1000 kg. Carnivores were classified according to three groups; small carnivores (e.g. fox); intermediate carnivore (e.g. wolf); and large carnivores (e.g. lion) (see Supplementary Table S3).

Faunal remains were also quantified by number of identifiable specimens (NISP) and minimum number of individuals (MNI). MNI estimates considered element laterality as well as their ontogenetic age^79^; epiphyseal fusion, long bone biometrics and, where applicable, dental wear. The age classes for mortality profiles were assigned to one of four different ages categories (infantile, juvenile, prime adult-adult and senile), based on tooth eruption and crown wear. For post-cranial specimens, epiphyseal fusion was considered.

Anatomical element profiles (Supplementary Table S2) were then organized into several anatomical regions; cranial (i.e. horn, cranium, mandible and teeth), axial (vertebrae, ribs, pelves and scapulae, according to^80^, upper appendicular elements (humerii, femora), intermediate appendicular limbs (radii, tibiae, patellae, ulnae), and lower appendicular elements (metapodials, carpals, tarsals, phalanges and sesamoids). Long limb bones were further divided into anterior portions (scapulae, humerii, radii, ulnae, carpals and metacarpals), as well as posterior portions (pelves, femora, tibiae, patellae, tarsals and metatarsals).

Several procedures were followed to reconstruct site formation processes, assess site integrity, as well as evaluate the contribution of various biogenic agents to the faunal assemblage. Bone fragmentation was assessed based on three variables. First, bones were divided into several categories according to their length: < 3 cm, 3.1–5.0 cm, 5.1–10 cm and > 10 cm (Table 3). Second, bones were classified according to whether they were fractured in green (fresh) or dry state^81^ (Table 3). Dry fractures are longitudinal and/or transverse to the axis of the bone as well, with uneven, rough and micro-stepped surfaces. Dry breaks also form with approximately right angles to the bone cortical surface. In contrast, specimens that are broken when fresh frequently have smoother and more obliquely-oriented fracture surfaces. Third, the percentage of shaft circumference has been defined following these categories; Type 1 are specimens that preserve < 25% of the shaft circumference intact; Type 2 are specimens with 25–50% of the shaft circumference; and Type 3 are specimens with > 50% of shaft circumference (Table 6).

The impact of fluvial activity was estimated with bone fragment size distributions and the presence of abrasion, polishing, rounded bones, and carbonates. Signs of polishing, rounding, or abrasion, are observed in transported assemblages, but also in non-transported assemblages exposed to circulating water and mobile sediments, such as those embedded in sand^82^. Weathering was assessed following^43^ (Table 3).

Bone surface modification analyses were carried out using 10–40 × magnification hand held lenses and binocular. Tooth marks were classified as pits, scores or punctures, while furrowing was also identified according to Refs.^83,84^. Modifications were quantified by specimen, with well-preserved bone surfaces based on NISP values. Carnivore activity can also be identified according to taphotypes^44^.

### Spatial analysis

Spatial analysis of VM4 consisted of three primary analyses, firstly testing for trends vertically on a palaeostratigraphic level, followed by analyses of horizontal distributions. Finally, assessments were performed for anisotropy and general orientation patterns across the site.

For palaeostratigraphy and the detection of discrete fossiliferous levels among the VM4 faunal assemblage, the artificially intelligent system proposed by Ref.^85^ was employed. This system uses unsupervised machine learning for pattern recognition, followed by Human-in-the-Loop supervision for interpretation, and finishing with the use of supervised machine learning for the fine-tuning of the final palaeostratigraphic model. For more details, please consult Supplementary Methods 1.

Once fossiliferous levels had been defined, spatial point patterns were analysed in detail across all levels of the VM4 site. These analyses included the calculation of density maps, hypothesis testing for Complete Spatial Randomness^86^, analyses of spatial correlation using both Ripley’s K-function and Besag’s L-function^87,88^, and finally the Hopkins–Skellam test^89^. To complement data revealed through statistical analyses, further use of unsupervised machine learning algorithms were performed to detect clusters across the horizontal axes of the site. For more details, please consult Supplementary Methods 2.

Finally, detailed statistical analyses were performed on orientation patterns through robust descriptive statistical analyses, as well as the construction of stereoplots for the combined visualisation of orientation and azimuth values. All statistical and data science applications were designed and implemented using the R programming language (v.3.5.1. 64-bit, https://www.r-project.org/). See Supplementary Methods 3 for further details.

## Supplementary Information

Supplementary Information.
