# Supplementary material for: Taphonomic and spatial analyses from the Early Pleistocene site of Venta Micena 4 (Orce, Guadix-Baza Basin, southern Spain)
Source: Sci Rep. 2021 Jul 7;11:13977. doi: 10.1038/s41598-021-93261-1 (PMC8263577; doi:10.1038/s41598-021-93261-1)
Supplement: Supplementary file 1 — Supplementary Information. [file 41598_2021_93261_MOESM1_ESM.docx]

**Supplementary Information**

**Taphonomic and spatial analyses from the Early Pleistocene site of Venta Micena 4 (Orce, Guadix-Baza Basin, southern Spain).**

Carmen Luzón, Jose Yravedra, Lloyd A. Courtenay, Juha Saarinen, Hugues-Alexandre Blain, Daniel DeMiguel, Suvi Viranta, Beatriz Azanza, Juan José Rodríguez-Alba, Darío Herranz-Rodrigo, Alexia Serrano-Ramos, Jose A. Solano, Oriol Oms, Jordi Agustí, Mikael Fortelius, Juan Manuel Jiménez Arenas.

**This document includes:**

**Supplementary Notes 1.** Stratigraphic sequence of Venta Micena and corresponding sites. – Page 2

Figure S1– Page 2

Figure S2– Page 3

**Supplementary Tables 1.** Microfauna Association from Venta Micena 1 and Venta Micena 2. – Page 4

Table S1a – Page 4

Table S1b – Page 4

**Supplementary Notes 2.** Macrovertebrates taxonomy. – Page 5

PROBOSCIDEA – Page 5

PERISSODACTYLA – Page 6

ARTIODACTYLA – Page 7

CARNIVORA – Page 10

**Supplementary Table S3.** Caption of species and relation to animal size – Page 18

**Supplementary Table S4.** Skeletal profiles tables. – Page 19

Table S4a – Page 19

Table S4b – Page 20

**Supplementary Methods.** Spatial Analysis. – Page 21

Supplementary Methods 1 – Page 21

Supplementary Methods 2 – Page 22

Supplementary Methods 3 – Page 23

Table S5 – Page 24

**Supplementary Notes 1. Stratigraphic sequence of Venta Micena and corresponding sites.**

**
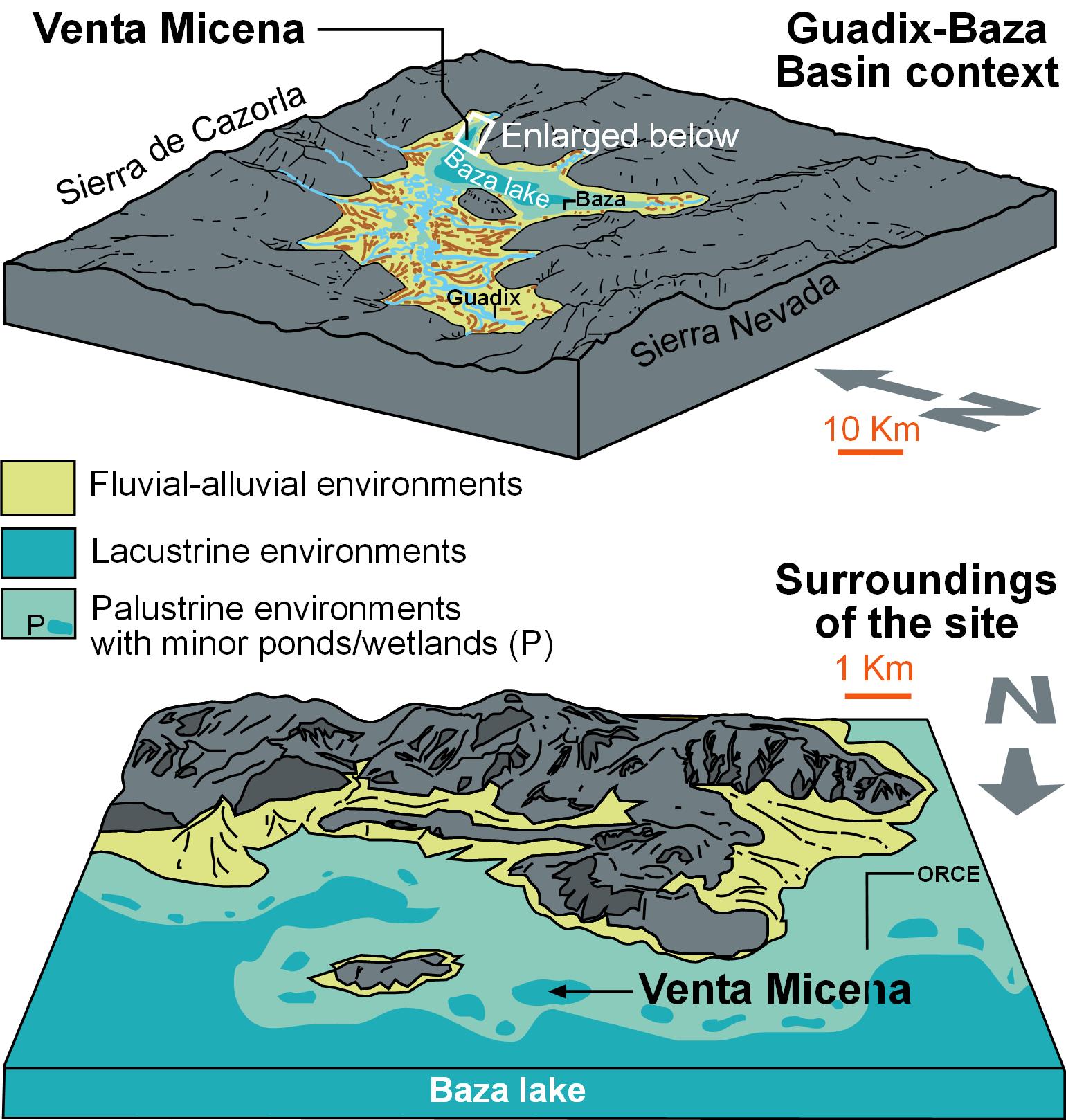
**

**Supplementary Figure S1**. Paleogeographic location of the Venta Micena site. Top: general context of the Guadix- Baza Basin, showing the main Baza lake. Bottom: surroundings of the site, with indication of the main Baza lake border and the palustrine areas including ponds such as those of Venta Micena (adapted and redrawn after many sources).

In the Venta Micena area, Anadón *et al.* (1986, 1987) described a sedimentary succession with 4 stratigraphic units (A to D). Unit C has a thickness ranging from 2.3 to 3.4 m and contains 3 different intervals (C0 to C2). C0 is characterized by a whitish sandy lutitic limestone while units C1 and C2 (known as Venta Micena limestone) are made by around 1.5m of micritic limestone of palustrine and lacustrine origin. C0 to C2 represent the same environmental conditions and are geologically of a similar age, as is also proved by biostratigraphical data (see main text). The VM fossil assemblages are located basically found in the lower part of the Venta Micena limestone (i.e., C1 interval), while in upper C2 interval, they are rather scarce. Geochemical data also display slight differences between C1 and C2 intervals (Granados *et al.,* subm).

Our detailed study on fossil distribution patterns at VM4 allows the identification of a lower VM4-I and an upper VM4-II sites, all found within the C1 interval. Previous studies of the VM3 site (360 m north of VM4) did not provide any detailed data on the vertical distribution of fossil remains. Because of this, its relative position has been marked by a dashed line in Sup. Fig. S2. The overlying D unit is a succession of muddy limestones interbedded with sandy marls, dolostones and marly dolostones of brownish and reddish colour (due to root bioturbation) with no presence of fossil bones.


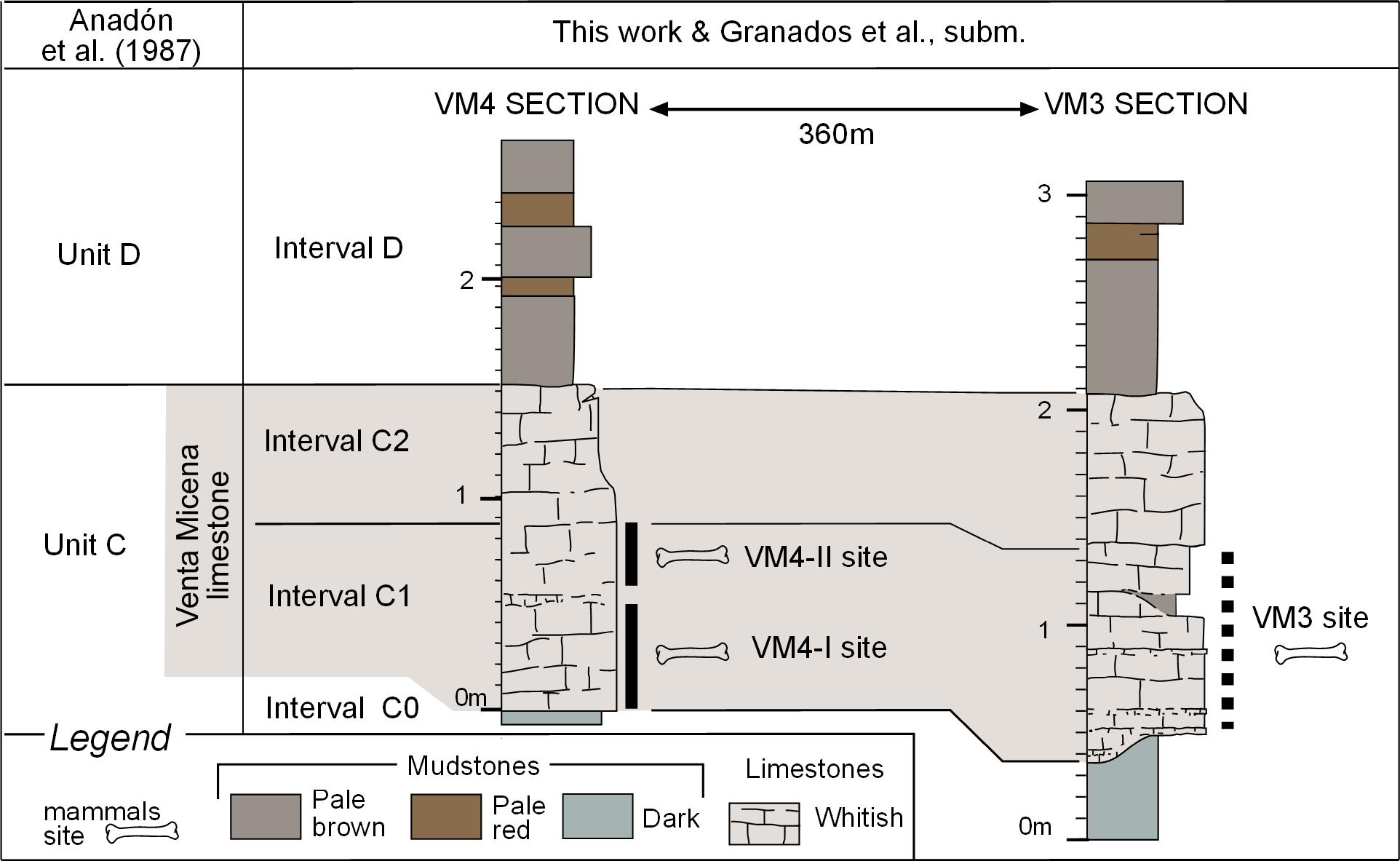


**Supplementary Figure S2**. Stratigraphic units A to D in VM4 section and site and VM4-1 and VM4-2.

**References cited in Supplementary Notes 1**

Anadón, P., Julià, R., De Deckker, E., Rosso, J. C. & Soulié‐Marsché, I. Contribución a la Paleolimnología del Pleistoceno inferior de la cuenca de Baza (sector Orce‐Venta Micena). *Paleontología i Evolució* **Memoria Especial 1**, 35‐72 (1987).

Anadón, P., De Deckker, P., & Julià, R. The Pleistocene Lake deposits of the NE Baza basin (Spain): salinity variations and ostracod succession. *Hydrobiologia* **143**, 199–208 (1986).

Granados, A., Oms, O., Anadón, P., Ibáñez-Insa, J., Kaakinen, A. & Jiménez-Arenas, J.M. Geochemical and sedimentary constraints on the formation of the Venta Micena Early Pleistocene site (Guadix-Baza basin, Spain). *Scientific Reports*, submitted.

**Supplementary Tables 1: Microfauna Association from Venta Micena**

| Class | Order | Family | Species/taxon |
| --- | --- | --- | --- |
| Amphibia | Anura | Alytidae | cf. *Discoglossus* sp. |
| Amphibia | Anura | Ranidae | *Pelophylax* *perezi* |
| Reptilia | Squamata | Lacertidae | Lacertidae indet. (cf. *Podarcis* sp.) |
| Reptilia | Squamata |  | Ophidia indet. |
| Mammalia | Eulipotyphla | Soricidae | *Asoriculus gibberodon* |
| Mammalia | Eulipotyphla | Talpidae | *Galemys* sp. |
| Mammalia | Rodentia | Cricetidae | *Allophaiomys pliocaenicus* |
| Mammalia | Rodentia | Cricetidae | *Allophaiomys ruffoi* |
| Mammalia | Rodentia | Gliridae | *Eliomys intermedius* |
| Mammalia | Rodentia | Muridae | *Apodemus* aff. *mystacinus* |
| Mammalia | Rodentia | Muridae | *Apodemus sylvaticus* |
| Mammalia | Rodentia | Muridae | *Castillomys crusafonti* |
| Mammalia | Lagomorpha | Leporidae | *Oryctolagus* cf. *lacosti* |
| Mammalia | Lagomorpha | Ochotonidae | *Prolagus calpensis* |

**Supplementary Table S1a.** Small mammals profile of Venta Micena 1

| Class | Order | Family | Species/taxon |
| --- | --- | --- | --- |
| Reptilia | Testudines | Testudinidae | *Testudo* sp. |
| Reptilia | Squamata | Lacertidae | Lacertidae indet. |
| Reptilia | Squamata |  | Ophidia indet. |
| Mammalia | Eulipotyphla | Talpidae | *Galemys pyrenaicus* |
| Mammalia | Rodentia | Cricetidae | *Allophaiomys ruffoi* |
| Mammalia | Rodentia | Gliridae | *Eliomys intermedius* |
| Mammalia | Rodentia | Hystricidae | *Hystrix major* |
| Mammalia | Rodentia | Muridae | *Apodemus* *mystacinus* |
| Mammalia | Rodentia | Muridae | *Apodemus* aff. *mystacinus* |
| Mammalia | Rodentia | Muridae | *Apodemus sylvaticus* |
| Mammalia | Rodentia | Muridae | *Castillomys crusafonti* |
| Mammalia | Lagomorpha | Leporidae | *Oryctolagus* cf. *lacosti* |

**Supplementary Table S1b.** Small mammals profile of Venta Micena 2

**References**

Agustí, J., Arbiol, S. & Martín-Suárez, E. Roedores y lagomorfos (Mammalia) del Pleistoceno inferior de la region de Orce (Granada, España). *Paleontologia i Evolucio* **Memoria especial 1**, 95–107 (1987).

Agustí, J., Oms, O., Garcés, M. & Parés, J.M. Calibration of the late Pliocene–Early Pleistocene transition in the continental beds of the Guadix-Baza basin (southeastern Spain). *Quaternary International* **40**, 93–100 (1997).

**Supplementary Notes S2. Macrovertebrates taxonomy.**

Table S2 lists the updated faunal list from all the sites of the Venta Micena limestone contains 40 taxa. The preceding lines explains these differences in detail.

| Class | Order | Family | Species |
| --- | --- | --- | --- |
| Mammalia | Carnivora | Canidae | *Xenocyon lycaonoides* (= *Lycaon lycaonoides*) |
| Mammalia | Carnivora | Canidae | *Canis mosbachensis* |
| Mammalia | Carnivora | Canidae | *Vulpes alopecoides* |
| Mammalia | Carnivora | Felidae | *Lynx* sp. |
| Mammalia | Carnivora | Felidae | *Homotherium latidens* |
| Mammalia | Carnivora | Felidae | *Megantereon cultridens* |
| Mammalia | Carnivora | Felidae | *Panthera* cf*. gombaszoegensis* |
| Mammalia | Carnivora | Hyaenidae | *Pachycrocuta brevirostris* |
| Mammalia | Carnivora | Ursidae | *Ursus etruscus* |
| Mammalia | Proboscidea | Elephantidae | *Mammuthus meridionalis* |
| Mammalia | Perissodactyla | Rhinocerotidae | *Stephanorhinus etruscus* |
| Mammalia | Perissodactyla | Equidae | *Equus altidens* |
| Mammalia | Artiodactyla | Hippopotamidae | *Hippopotamus antiquus* (= *H. major*) |
| Mammalia | Artiodactyla | Cervidae | Medium-sized cervid  (“*Cervus” elaphoides,* Cervidae indet., *Pseudodama* sp. or *Metacervoceros rhenanus* |
| Mammalia | Artiodactyla | Cervidae | Very large-sized cervid  (*Praemegaceros* *solilhacus, Megaceroides* aff. *solilhacus, Eucladoceros giulii, Praemegaceros* cf. *P. verticornis, Eucladoceros* sp.) |
| Mammalia | Artiodactyla | Bovidae | *Capra alba* (= *Hemitragus albus*) |
| Mammalia | Artiodactyla | Bovidae | *Soergelia minor* |
| Mammalia | Artiodactyla | Bovidae | *Praeovibos* sp. |
| Mammalia | Artiodactyla | Bovidae | *Bison* sp. |
| Mammalia | Artiodactyla | Bovidae | *Hemibos* aff*. gracilis* |
| Mammalia | Artiodactyla | Bovidae | Caprini indet. (small, *Rupicapra*-sized) |

**Supplementary Table S2.** Macrovertebrates profile of Venta Micena

PROBOSCIDEA

**Elephantidae**

*Mammuthus meridionalis* Nesti, 1825

Proboscideans are represented by a single species, the “ancestral” or “southern” mammoth (*Mammuthus meridionalis*). The material described so far from Venta Micena includes mostly teeth and cranial material of juvenile individuals, including isolated deciduous premolars (DP2 and DP3), skull fragments and a juvenile skull with left and right DP2 and DP3 (VM-3662) (Ros-Montoya, 2010; Ros-Montoya *et al.*, 2012). In addition, there is a mandible of an old adult individual with posterior part of left m3 (VM-4103) (Ros-Montoya, 2010; Ros-Montoya *et al*., 2012). These materials can be confidently identified as *M. meridionalis* based on dental and cranio-mandibular characteristics, such as enamel thickness, lamellar frequency, degree of enamel plication and general morphology of the (rather poorly preserved) juvenile skull and adult mandible (see comparisons in Ros-Montoya (2010) and Ros-Montoya *et al.* (2012)).

New specimens of *M. meridionalis* were discovered in VM 4 during the field season of 2018. These include associated left DP2 and DP3 milk premolars (IDs 182000282 and 182000281, respectively), diaphysis fragment of a left femur (ID 182000675) and an articulated set of eight vertebrae, starting from 2^nd^ cervical (axis) (ID 182000810).

PERISSODACTYLA

**Rhinocerotidae**

*Stephanorhinus etruscus* Toula, 1902

The rhinoceros remains from Venta Micena were originally assigned to *Dicerorhinus* (today *Stephanorhinus*) *etruscus* (Agustí, 1987). Santafe-Llopis & Casanovas-Cladellas (1987) attributed them to the subspecies *Dicerorhinus etruscus brachycephalus* erected by Guérin (1980). As explained in Fortelius *et al.* (1993), this subspecies was unfortunately based on a specimen from Daxlanden that actually represents *“Stephanorhnius” kirchbergensis* and is therefore deeply problematic. Guérin’s attempt, to formally recognise the early and late morphotypes of “*Dicerorhinus etruscus*”, was considered valid by Fortelius *et al.* (1993), who proposed using already available species-level names for them, *Stephanorhinus etruscus* and *Stephanorhinus hundsheimensis* respectively. Since the intended concept of Guerin’s nomenclature *Dicerorhinus etruscus brachycephalus* essentially maps onto *Stephanorhinus hundsheimensis,* this name, usually denoted with a cautionary “cf.”, has more or less automatically been applied to the Venta Micena rhinoceros in recent decades (Martínez Navarro *et al*., 2014; Rodríguez-Gómez *et al.,* 2017; Palmqvist *et al.,* 2018). This is surprising because elsewhere the species is typical of the Middle rather than Early Pleistocene.

Species-level identification of the Venta Micena rhinoceros:

Teeth are unfortunately unhelpful for distinguishing between *S. etruscus* and *S. hundsheimensis* (Fortelius *et al*., 1993). Nevertheless skulls and many postcrania are diagnostic. Without undertaking a full review of the large collection of rhinoceros remains from Venta Micena, the species attribution is based on a few diagnostic specimens. These include the skull (VM-3667) currently on display as part of a larger collection of fossils in exhibition at the Museo de los Primeros Pobladores de Europa ‘Josep Gibert’ and a talus bone retrieved during the 2019 field season (ID 192000186).

The skull is damaged and deformed but quite complete. It is slender and the facial portion is of modest size, not expanded as in *S. hundsheimensis.* The anterior rim of the orbit is above M^1^ rather than above M^2^ as is typical for *S. hundsheimensis*. The narial incision is relatively short, reaching back only above the P^3^/P^4^ contact, rather than P^4^/M^1^ as in *S. hundsheimensis* (Fortelius *et al.,* 1993).

The talus bone is very small, with a maximum length of only 68 mm, compared with the mean values of 83 mm in *S. hundsheimensis* and 73 mm in *S. etruscus* (Fortelius *et al*., 1993). Other dimensions are similarly in the low range of *S. etruscus*.

Based on the specimens seen during a recent examination of the Venta Micena collections, at this moment in time there is no reason to challenge the proposed identification, at least until further controls can be run or new fossil remains found. From this perspective, the rhinoceros materials have been attributed to *S. etruscus*.

**Equidae**

*Equus altidens* von Reichenau, 1915

The abundant fossil remains of equids from Venta Micena have all been assigned to the stenonid *Equus altidens*. The equid material from Venta Micena was originally described as a new subspecies of *Equus stenonis*, *E. s. granatensis*, which was distinguished from other “subspecies” of *E. stenonis* by its smaller size and gracile limb bones (Alberdi and Ruiz Bustos 1985). In a revision by Guerrero-Alba and Palmqvist (1997) the equid from Venta Micena was re-assigned into the species *Equus altidens* based on morphometric comparison with the type material from Süssenborn, Germany. In a revision of European stenonid horses (Alberdi *et al*., 1998), the material from Venta Micena was also identified as *Equus altidens* based on typical stenonid dental characteristics (relatively short protocone in upper molars and strong ectoflexid and V-shaped linguaflexid in lower molars) combined with smaller size and more gracile limb bones than in other more robust stenonid species. Nevertheless, the authors considered this material to belong to a different subspecies *E. a. granatensis* based on its smaller size, shorter metapodials and more pronounced distal part of the protocone in the upper molars (Alberdi *et al.*, 1998). After the original descriptions, all the following occurrences of the abundant equid fossils from Venta Micena have been identified as *E. altidens* (e.g., Palmqvist *et al.,* 1996, 2003, 2018; Rodríguez-Gómez *et al*., 2017).

ARTIODACTYLA

**Hippopotamidae**

*Hippopotamus antiquus* Desmarest, 1822

The hippopotamus material from Venta Micena has been assigned to the common Early Pleistocene European species *Hippopotamus antiquus* (= *H. major* Cuvier 1824) based mainly on typical dental characteristics of the genus and relatively large size in comparison to the extant (and Late Pleistocene European) *Hippopotamus amphibius* (Alberdi and Ruiz Bustos, 1985). Molar sizes in the Venta Micena *Hippopotamus* is comparable to those of other Early Pleistocene European populations of similar age (Alberdi and Ruiz Bustos, 1985).

**Cervidae**

The taxonomic identification of the Venta Micena cervids has been problematic. This is largely due to the fact that at Venta Micena antlers, which are the most diagnostic feature of these ruminants, are rare and fragmentary. Consequently, although numerous, cervid teeth and postcranials do not allow reliable taxonomic assignments. An additional difficulty is that the systematics of the European Early Pleistocene deer species are still unsettled and authors have not yet come to a consensus on the various genera and lineages. VM4 provides new remains whose taxonomical identity is being verified. What is clear is that there are two different sized cervids. Recently, Martinez-Navarro *et al.* (2016) and Rodríguez-Gómez *et al*. (2017) reported the possible presence of a still undetermined small-sized deer, but this is yet to be confirmed.

Medium-sized cervid

[“*Cervus” elaphoides* Kalkhe, 1960, Cervidae indet., *Pseudodama* sp., or *Metacervoceros rhenanus* (Dubois, 1904)]

Menéndez (1987) originally assigned the medium-sized cervid from VM3 to the species “*Cervus” elaphoides* Kahlke 1960 (at that time considered to be commonly present in Western and Central Europe mid-Pleistocene localities), and distinguished it from both, the three-tined *Metacervocerus rhenanus*, also present in Western and Central Europe, and the four-tined *Pseudodama/Cervus nestii* from the Italian peninsula. Nevertheless, Lister (1990) shows that “*Cervus” elaphoides* remains were actually juvenile antlers of *Cervus elaphus acoronatus*, and that other antlers, teeth and postcranial remains from Mosbach belonged to a representative of the fallow deer lineage. Kahlke (1995) renamed them *Cervus* s.l. *reichenaui*. Lister (1990) suggested that the smaller cervid of VM3 represents a yet unnamed medium-sized species, and Kahlke (1997) agreed to leave it in open nomenclature. Later on, it appeared in the new faunal list of the site indicated as Cervidae indet. (Palmqvist *et al*., 1996; Arribas and Palmqvist, 1998) or as *Pseudodama* sp.

Martínez-Navarro and colleagues (2004), followed Azzaroli (1992), who created the latter genus, as a means of including all these fallow deer-sized cervids. Abbazzi (2010) excluded any relationship with the Italian *Pseudodama* lineage, and considered it to be conspecific with the medium-sized deer, present in the near sites of Fuente Nueva 3 and Barranco León 5, which they refer to as *Metacervocerus rhenanus.* Indeed, noting dental similarities to *Metacervocerus rhenanus*, Croitor *et al*. (2018) called the medium-sized cervid from VM3 *’Cervus elaphoides’*, considering it a putative forerunner of the endemic Iberian lineage leading to *Haploidoceros*. VM4 provided a much more complete antler than those available from VM3. Although severely damaged, the very open basal fork is inserted above the burr (at a distance approximately equivalent to twice the diameter of the shaft). At this level, the beam first bends back and then again curves markedly upwards and also laterally, without accessory knob.

Very large-sized cervid

[*Praemegaceros* *solilhacus* (Robert, 1829)*, Megaceroides* aff. *solilhacus* (Robert, 1829)*, Praemegaceros* cf. *verticornis* (Dawkins, 1872), *Eucladoceros giulii* Kahlke, 1995, *Eucladoceros* sp.]

Fossils of very large-sized cervids from VM3 were originally identified as *Praemegaceros solilhacus* by Menéndez (1987), based mainly on dental and antler-base morphology. Abbazzi and Masini (1997) assigned them to *Megaceroides* aff. *solilhacus* basing on cranial traits and pedicle orientation, as well as on the slenderness of the limb bones. Abbazzi (2010) ignored the higher position of the first bifurcation and the divergent beam morphology when she stated that the larger deer of VM3 is conspecific with the large-sized deer present in the near sites of Fuente Nueva 3 and Barranco León 5 which she attributed to *Praemegaceros verticornis.* Nevertheless, she noticed that the size and proportions of the postcranial bones in the VM3 sample are different from those of equivalent bones of *P. verticornis.* Abbazzi (2010) was followed by other scholars who assigned the larger deer from Venta Micena to *Praemegaceros* cf. *verticornis* (Rodríguez-Gómez *et al*., 2017; Palmqvist *et al*., 2018). Another point of view is that of Kahlke (1997) and Van der Made (1998, 1999, 2001). Because of the long metapodials, Kahlke (1997) considered the larger deer of VM3 to be conspecific with *Eucladoceros giulii* from Untermassfeld (Early Pleistocene, Germany). Following this view, Van der Made (1998, 1999, 2001) assigned to this species many large cervid materials from several iberian Early Pleistocene sites, including Venta Micena. Nevertheless, Croitor and Kostopoulos (2004) excluded the Venta Micena material from this controversial species (that should be named as *Rucervus (Arvernoceros) giulii,* according to Croitor, 2018) following Abbazzi and Massini (1997). Recently, Made and Dimitrijevic (2017) left the VM3 large deer in open nomenclature as *Eucladoceros* sp. suggesting its closer relationship with *E. montenegrensis* that they described from Trlica (Early Pleistocene, Montenegro).

Two fragments of shed antlers from VM4 (IDs 182000833 and 182000496), the first one more complete than the VM3 specimens, show the basal morphologic traits described by Menéndez (1987). In particular, the basal bifurcation positioned high above the burr and the absence of accessory knobs or of a spurious basal time are suggestive of *Praemegaceros solilhacus*. Also the morphology of the long second segment of the beam excludes it from *P. verticornis*.

Further analyses of the new material will undoubtedly provide more information on the identity of the cervid species. Therefore, and taking a conservative approach, we will for the moment keep the denomination *P.* cf. *verticornis* following the criteria of previous research teams in Orce.

**Bovidae**

**Caprini**

*Capra alba* (*= Hemitragus albus*) Moyà-Solà, 1987

*Capra alba* was first described from Venta Micena by Moyà-Solà (1987) as a plesiomorphic species of *Capra* with distinct horn-core morphology. The horn-cores are laterally compressed, keeled and oval in cross-section at the base, and relatively upright and gently backwards-bent without torsion. The presence of larger, more robust horn-cores alongside smaller and more slender ones, suggests a strong sexual dimorphism typical of the genus *Capra*. This species was originally described as *Hemitragus* sp. by Moyà-Solà *et al*. (1981) and assigned to that genus by Crégut-Bonnoure (1999), based on specific dental and postcranial characteristics. Nonetheless, a recent revision of the horn-core morphology and dimorphism, as well as of the postcranial morphology, supports the inclusion of this species in the genus *Capra* (van der Made *et al*., 2008). Morphologically, the postcranial of this species is similar to those of *Capra ibex* and *C. pyrenaica*, but more gracile (Moyà-Solà, 1987; van der Made *et al*., 2008). Van der Made *et al*. (2008) interpreted the difference as a less derived adaptation toward mountainous habitats than that of modern species of *Capra* and *Hemitragus*.

Caprini indet., small sp.

The presence of an unidentified small-sized caprine bovid in Venta Micena is indicated by a posterior metatarsal fragment much smaller than those of *Capra alba* and similar in size to those of the extant *Rupicapra* (Moyà-Solà, 1987).

**Ovibovini**

*Soergelia minor* Moyà-Solà, 1987

The smaller, frequently found ovibovine bovid from Venta Micena was described as a new species *Soergelia minor* by Moyà-Solà (1987), based on less derived morphological characteristics than in the type species *S. elisabethae* from the Middle Pleistocene of Süssenborn, Germany. These include smaller sized, less derived dentition (shorter m3, longer premolar row and overall less hypsodont dentition), and more primitive horn-core shape (strongly diverging and forward-oriented, but narrower and less laterally and ventrally curving than in the type species *S. elisabethae*). The postcranial bones are more slender than in *S. elisabethae*.

*Praeovibos* sp. Staudinger, 1908

The remains of a larger ovibovine *Praeovibos* sp. are very scarce at Venta Micena, but the presence of this species is clearly demonstrated by characteristic horn-core and postcranial material (Moyà-Solà, 1987). The horn-core morphology is typical of *Praeovibos*, characteristically downwards-bent and with forward-bending tip and distinct ventral grooves. The metapodials are larger and more robust than in *Soergelia minor*, but less so than in the Middle Pleistocene *P. priscus*.

**Bovini**

*Bison* sp. Linnaeus, 1758

The abundant large bovine bovid from Venta Micena was originally described as *Bison* sp., based on dental characteristics and marked postcranial sexual dimorphism (Moyà-Solà, 1987). The bison from Venta Micena is similar to other Early Pleistocene bisons (e.g. *Bison palaeosinensis, B. tamanensis, B. georgicus* and *B. degiulii*). It is smaller, less robust, and has relatively simpler dentition than the Middle-Late Pleistocene *B. priscus*. There is remarkable sexual dimorphism in the size and robustness of the postcranial bones. A partial subadult skull roof with incomplete left horn-core (VM-8000) of *Bison* sp. was described by Martínez-Navarro *et al*. (2011). This specimen shows highly pneumatized frontal bones and gently upwards-curving horn-core typical of the genus *Bison*.

*Hemibos* sp. Rutimeyer, 1865, *H.* aff*. gracilis*

A partial skull roof with fragmentary horn-core bases (VM-9000) from Venta Micena has been described as *Hemibos* aff. *gracilis* (Martínez-Navarro *et al*., 2011). The horn-cores are robust, keel-less and dorso-laterally oriented. Martínez-Navarro *et al*. (2011) considered the orientation of the horn-cores, similar to that of *Hemibos gracilis* from the Early Pleistocene of China. So far, this is the only specimen from Venta Micena that has confidently been assigned into *Hemibos*, but Martínez-Navarro *et al*. (2011) suggest that the high variation in size and robustness of large bovine postcranials from Venta Micena could be partly due to the presence of *Hemibos*, and not reflect only the sexual dimorphism of the more abundant *Bison* sp.

CARNIVORA

**Canidae**

*Xenocyon* (*Lycaon*) *lycaonoides* Kretzoi, 1938

The taxonomy of the Venta Micena canids has been controversial as is for, in general, all Pleistocene *Canidae.* Two species are currently recognized at Venta Micena (e.g. Medin *et al*., 2017), instead of the three identified originally by Pons-Moya (1987). The assignments are supported morphologically as well as by isotopically-based ecological data.

The larger and apparently hypercarnivorous species is represented by abundant cranial and postcranial material (Palmqvist *et al*., 1999), and is assigned to *Xenocyon lycaonoides*, a wild dog-like canid widespread in Europe during the Pleistocene. The species is placed in the extinct genus *Xenocyon* following the recent works by Hartstone-Rose *et al*. (2010), Wang *et al*. (2015) and Zrzavý *et al*. (2018). Hartstone-Rose *et al*. (2010) described a skeleton from South Africa (1-1.9 Ma) with mosaic of characters linking it to the modern *Lycaon,* for according to the latter authors *Xenocyon* evolved in Africa. Wang *et al*. (2015) analysed the Old World Pleistocene hypercarnivorous canid lineages and found four independently evolving lines; *Xenocyon*, *Cuon*, *Sinicuon* and *Lycaon*. The phylogenetic analysis by Zrzavý *et al*. (2018) ruled out any close affinity between *Lycaon* and *Xenocyon*. The two genera differ, for instance, in the morphology of the paranasal sinuses and as well as in dental characters.

*Canis mosbachensis* Soergel, 1925

*C. mosbachensis* is a medium sized canid, often referred to as jackal-like ecologically speaking. *C. mosbachensis* is probably an early member, a chrono species that can be counted as part of the modern Holarctic gray wolf *C. lupus* – clade.  *C. lupus* today shows remarkable variation in size and shape throughout its range. Recently, it has been suggested that the Venta Micena Canis represents a new taxon, *Canis orcensis* (Martínez-Navarro *et al.*, 2021).

*Vulpes alopecoides* Kormos, 1932

Small carnivores are less well known from Venta Micena. Several canid postcranial bones as well as teeth of the size of *Vulpes praeglacialis* have been found. According to Lucenti & Madurell-Malapiera (2020), the early Pleistocene *Vulpes* in Europe belong to *V. alopecoides* for which the *V. praeglacialis* is a junior synonym (Bartolini-Lucenti & Madurell-Malapiera, 2020).

**Ursidae**

*Ursus etruscus* Cuvier, 1823

A bear taxon, *Ursus etruscus,* is identified from Venta Micena and recently been studied by Medin *et al*. (2017). *U. etruscus* is represented by at least 3 adults and a juvenile from the site. The adults are represented by crania, teeth and some postcranial material and the juvenile by cranial material (Medin *et al.,* 2017 and unpublished).

**Felidae**

**Machairodontinae**

*Megantereon cultridens* Cuvier, 1824

Based on a comparative odontometrical analysis, Martínez Navarro & Palmqvist (1995) found that the Early Pleistocene specimens of *Megantereon* from Venta Micena show higher morphometric affinity with *M. whitei* than with *M. cultridens*. They therefore assigned the Venta Micena 3 *Megantereon* to the African *M. whitei.* Nevertheless, Turner (1987) thought the dimensional and anatomical variability of M*egantereon* fall in the range of sexual dimorphism and geographic variation. A later morphometric study by Palmqvist *et al*. (2007) assigned the European Calabrian *Megantereon* to *M. whitei*. A recent analysis by Lewis and Werdelin (2010) attributes the European Early Pleistocene *Megantereon to* *M. cultridens*. Dental characters are not diagnostic for taxonomic assignment at the species level.

*Homotherium latidens* Owen, 1846

*Homotherium latidens* is the only member of the species known so far in Europe (Turner & Antón, 1996). Palmqvist et al (1996) identified the remains of two individuals in VM3. In VM4, this species is only represented by a tooth fragment.

**Felinae**

*Lynx pardinus* Temminck, 1927

The Venta Micena lynx represents the modern Iberian lynx, *Lynx. pardinus*. According to the review by Boscaini *et al*. (2015) *L. pardinus* appeared in the Iberian fossil record between 1.6 nd 1.7 M. *L. pardinus* is represented by at least one individual in Venta Micena 3 (Palmqvist *et al*., 1996).

**Pantherine**

*Panthera cf. gombaszoegensis* Kretzoi, 1938

According to Marciszak (2014) and Rodríguez-Gómez *et al*. (2017). *Panthera gombaszoegensis* is present in the Venta Micena 3 assemblage, but is not observed in Venta Micena 4.

**Hyaenidae**

*Pachycrocuta brevirostris* Aymard, 1846.

The only hyaenid of Venta Micena is the European Pliocene to Middle Pleistocene*, Pachycrocuta. brevirostris* (Palmqvist *et al*., 1996; 2011). This hyaenid has been considered as the main bone accumulation agent at Venta Micena 3. In fact the assemblage has been interpreted as a bone accumulation typical of a *Pachycrocuta* den (Palmqvist & Arribas, 2001).

**References**

- Abbazzi L. An overview on *Praemegaceros* species from Pleistocene Italian localities, and remarks on the validity of the generic name *Praemegaceros* Portis 1920. *Atti dell'Accademia Nazionale dei Lincei* s. 9, 15, 115-132 (2004).
- Abbazzi, L. La fauna de cérvidos de Barranco León y Fuente Nueva 3 in *Ocupaciones Humanas en el Pleistoceno inferior y medio de la cuenca de Guadix-Baza, Memoria Científica* (eds. Toro, I., Martínez-Navarro, B.& Agustí, J.) 273-290 (Junta de Andalucía. Consejería de Cultura. E.P.G. Arqueología Monográfico, 2010.
- Abbazzi, L., Masini, F. *Megaceroides solilhacus* and other deer from the middle Pleistocene site of Isernia la Pineta (Molise, Italy). *Bollettino della Società Paleontologica Italiana* 35, 213-227 (1997).
- Agustí, J., Lozano-Fernández, I., Oms, O., Piñero, P., Furió, M., Blain, H.-A., López-García, J.M. & Martínez-Navarro, B. Early to Middle Pleistocene rodent biostratigraphy of the Guadix-Baza Basin (SE Spain). *Quaternary International* 389, 139-147 (2015).
- Agustí, J. Introducción: Historia del yacimiento de Venta Micena. *Paleontologia i Evolució* Memoria Especial 1, 3-9 (1987).
- Alberdi, M.T. & Ruiz-Bustos, A. Descripción y significado bioestratigráfico del *Equus* e Hipopótamo en el yacimiento de Venta Micena (Granada). *Estudios Geológicos* 41, 251-261 (1985).
- Alberdi, M.T., Ortiz-Jaureguiz, E. & Prado, J.L. A quantitative review of european stenonid horses. *Journal of Paleontology.* 72, 371-387 (1998).
- Arribas, A. & Palmqvist, P. El modelo de actividad de *Pachycrocuta* en Venta Micena: implicación de los hiénidos en el registro paleomastológico Plio‐Pleistoceno español. *Temas Geológico‐Mineros* 26, 173‐181 (1999).
- Arribas, A. & Palmqvist, P. Taphonomy and palaeoecology of an assemblage of large mammals: hyaenid activity in an Early Pleistocene site at Spain. *Geobios* 31, (Supplement 3), 3-47 (1998).
- Azzaroli, A. The cervid genus *Pseudodama* n. g. in the Villafranchian of Tuscany. *Palaeontographia Italica* 79, 1–41 (1992).
- Bartolini-Lucenti, S. B. & Madurell-Malapiera, J. Unraveling the fossil record of foxes: An updated review on the Plio-Pleistocene Vulpes spp. from Europe. *Quaternary Science Reviews* 236, 106296 (2020).
- Boscaini, A., Madurell-Malapiera, J., Llenas, M. & Martínez-Navarro, B. The origin of the critically endangered Iberian lynx: Speciation, diet and adaptive changes. *Quaternary Science Reviews* 123, 247-253 (2015).
- Breda, M. & Lister, A.M. *Dama roberti*, a new species of deer from the early Middle Pleistocene of Europe, and the origins of modern fallow deer. *Quaternary Science Reviews* 69, 155-167 (2013).
- Breda, M., Peretto, C. & Thun Hohenstein, U. The deer from the early Middle Pleistocene site of Isernia la Pineta (Molise, Italy): revised identifications and new remains from the last 15 years of excavation. *Geological Journal* 50/3, 290-305 (2015).
- Crégut-Bonnoure, E. Les petits Bovidae de Venta Micena (Andalousie) et de Cueva Victoria (Murcia) in *Proceedings of the International Conference of Human Paleontology* (eds. J. Gibert *et al*.) 191-228 (Orce, 1999).
- Croitor, R, Bonifay, M.F. & Brugal, J.P. Systematic revision of the endemic deer *Haploidoceros* n. gen. *mediterraneus* (Bonifay, 1967) (Mammalia, Cervidae) from the Middle Pleistocene of Southern France. *Paläontologische Zeitschrift* 82(3), 325–346 (2008).
- Croitor, R. & Kostopoulos, D.S. On the systematic position of the large sized deer from Apollonia, Early Pleistocene, Greece. *Paläontologische Zeitschrift* 78 (1), 137-159 (2004).
- Croitor, R. Early Pleistocene small-sized deer of Europe. *Hellenic Journal of Geosciences* 41 (1), 89-117 (2006).
- Croitor, R., Sanz, M. & Daura, J. The endemic deer *Haploidoceros mediterraneus* (Bonifay) (Cervidae, Mammalia) from the Late Pleistocene of Cova del Rinoceront (Iberian Peninsula): origin, ecomorphology, and paleobiology. *Historical Biology* 32:3, 409-427 (2018)
- de Vos, J., Mol, D. & Reumer, J.W.F. Early Pleistocene Cervidae (Mammalia, Artiodactyla) from the Oosterschelde (the Netherlands), with a revision of the cervid genus *Eucladoceros* Falconer, 1868. *Deinsea* 2, 95-121 (1995).
- Di Stefano, G. & Petronio, C. Systematics and evolution of the Eurasian Plio-Pleistocene tribe Cervini (Artiodactyla, Mammalia). *Geologica Romana* 36, 311–334 (2003).
- Fortelius, M., Mazza, P. & Sala, B. *Stephanorhinus* (Mammalia:Rhinocerotidae) of the Western European Pleistocene, with a revision of *S. etruscus* (Falconer, 1868). *Palaeontographia Italica* 80, 63–155 (1993).
- Garrido, G. Paleontología sistemática de grandes mamíferos del yacimiento del Villafranquiense Superior de Fonelas P-1 (Cuenca de Guadix, Granada). (*Tesis Doctoral, Universidad Complutense de Madrid,* 2006).
- Garrido, G. Presencia de *Mammuthus meridionalis* (Nesti, 1825) en el Villafranquiense superior de Fonelas P-1 (Cuenca de Guadix, Granada) in *Vertebrados del Plioceno superior terminal en el suroeste de Europa: Fonelas P-1 y el Proyecto Fonelas* (ed. Arribas, A.) 597-607 (Cuadernos del Museo Geominero, 2008).
- Guérin, C. Les rhinocéros (Mammalia, Perissodactyla) du Miocèneterminal au Pléistocène supérieur en Europe occidentale, comparaison avec les espèces actuelles. *Documents des laboratoires de géologie de la Faculté des Sciences de Lyon* 79, 3–1183 (1980).
- Guerrero-Alba, S. & Palmqvist, P. Morphometric study of the horse from Venta Micena (Orce, Granada) and its comparison with both extant and Plio-Pleistocene equids from Europe and Africa. *Paleontologia i Evolució* 30–31, 93–148 (1997).
- Hartstone-Rose, A., Werdelin, L., De Ruiter, D. J., Berger, L. R., & Churchill, S. E. The Plio-Pleistocene ancestor of wild dogs, *Lycaon sekowei* n. sp. *Journal of Paleontology*, 84(2), 299-308 (2010).
- Heintz, E. Les cervidés villafranchiens de France et d’Espagne. *Mémoires du Muséum National d’Histoire Naturelle, N. S., série C, Sciences de la Terre* 22, 1–303 (1970).
- Kahlke, H.-D. Die Cerviden-Reste aus dem Unterpleistozän von Untermassfeld in *Das Pleistozän von Untermassfeld bei Meiningen (Thüringen)* (ed. Kahlke, R.D.) 181-275 (Dr Rudolf Habelt GMBH, Bonn, 1997).
- Kahlke, H.-D. Die Cerviden-Reste aus den altpleistozänen Sanden von Mosbach (Biebrich-Wiesbaden), teil 1, Die Gewerhe, Gehorne und Gebisse. *Abhandlungen der Deutschen Akademie der Wissenschaften zu Berlin* 7, 1-75 (1960).
- Kahlke, H.-D. Ein fossiler Elch-Fund aus dem Unterpleistozän von Untermaßfeld in Thüringen (Mitteldeutschland) *Quartär* 45/46, 227-235 (1995),
- Lister, A.M. Critical reappraisal of the Middle Pleistocene deer species "*Cervus*" *elaphoides*. *Quaternaire* 3-4,175-192 (1990).
- Lewis, M. & Werdelin, L. Carnivoran Dispersal Out of Africa During the Early Pleistocene: Relevance for Hominins? *Out of Africa* I, 13-26 (2010).
- Made, J. van der, Dimitrijevic, V. Eucladoceros montenegrensis n. sp. and other Cervidae from the Early Pleistocene of Trlica (Montenegro). *Quaternary International* 389, 90-118 (2015).
- Made, J. van der, Tong, H.W. Phylogeny of the giant deer with palmate brow tines *Megaloceros* from west and *Sinomegaceros* from east Eurasia. *Quaternary International* 179, 135-162 (2008).
- Made, J. van der. Ungulates from Gran Dolina (Atapuerca, Burgos, Spain). *Quaternaire* 9 (4), 267-281 (1998).
- Maglio, V.J. Origin and evolution of the Elephantidae. *Transactions of the American Philosophical Society* 63, 1–149 (1973).
- Marciszak, A. Presence of *Panthera gombaszoegensis* (Kretzoi, 1938) in the late Middle Pleistocene of Biśnik Cave, Poland, with an overview of Eurasian jaguar size variability. *Quaternary International* 326, 105-113 (2014).
- Martínez‐Navarro, B. Hippos, pigs, bovids, sabertoothed tigers, monkeys and hominids dispersals during late Pliocene and Early Pleistocene times through the levantine corridor in *Human Paleoecology in the Levantine Corridor* (eds. Goren‐Inbar, N., Speth, J. D.) 37‐51 (Oxbow Books, 2004).
- Martínez-Navarro, B. & Palmqvist, P. Presence of the African machairodont *Megantereon whitei* (Broom, 1937) (Felidae, Carnivora, Mammalia) in the Early Pleistocene site of Venta Micena (Orce, Granada, Spain), with some considerations on the origin, evolution and dispersal of the genus. *Journal of Archaeological Science* 22(4), 569-582 (1995).
- Martínez-Navarro, B., Espigares, M.P., Pasto, I., Ros-Montoya, S., & Palmqvist, P. Europe: early Homo fossil records in *Encyclopedia of Global Archaeology, vol. 1.* (ed. Smith, C.) 2561-2570 (Springer, 2014).
- Martínez-Navarro, B., Ros-Montoya, S., Espigares, M. & Palmqvist, P. Presence of the Asian origin Bovini, *Hemibos* sp. aff. *Hemibos gracilis* and *Bison* sp., at the Early Pleistocene site of Venta Micena (Orce, Spain). *Quaternary International* 243(1), 54-60 (2011).
- Martinez-Navarro, B., Bartolini Lucenti, S., Palmqvist, P., Ros-Montoya, S., Madurell-Malapiera, J., & Espigares, M.P. A new species of dog from the Early Pleistocene site of Venta Micena (Orce, Baza Basin, Spain). *Comptes Rendus Palevol* 20, 297-314 (2021).
- Medin T., Martínez-Navarro, B., Rivals, F., Madurell-Malapiera, J., Ros-Montoya, S., Espigares, M., Figueirido, B., Rook, L. & Palmqvist, P. Late Villafranchian *Ursus etruscus* and other large carnivorans from the Orce sites (Guadix-Baza basin, Andalusia, southern Spain): Taxonomy, biochronology, paleobiology, and ecogeographical context. *Quaternary International* 431, 20-41 (2017).
- Menéndez, E. Cérvidos del yacimiento del Pleistoceno inferior de Venta Micena 2, Orce (Granada, España). *Paleontologia i Evolució* Memoria Especial 1, 129-180 (1987).
- Moyà Solà, S., Agustí, J., Gibert, J. & Vera. J.A. (eds.) *Geología y Paleontología del Pleistoceno inferior de Venta Micena. Paleontologia i Evolució* Memoria Especial 1 (1987).
- Moyà-Solà, S., Agustí, J., Gibert, J. & Pons-Moyà, J. El yacimiento cuaternario de Venta Micena (España) y su importancia dentro de las asociaciones faunísticas del Pleistoceno inferior europeo. *Paleontologia i Evolució* 16, 39-53 (1981).
- Palmqvist, P. & Arribas, A. Taphonomic decoding of the paleobiological information locked in a Early Pleistocene assemblage of large mammals. *Paleobiology* 27(3), 512-530 (2001).
- Palmqvist, P., Martínez-Navarro, B., Pérez-Claros, J.A., Torregrosa, V., Figueirido, B., Jiménez-Arenas, J.M., Espigares, M., Ros-Montoya, S. & De Renzi, M. The giant hyena *Pachycrocuta brevirostris*: Modelling the bone-cracking behavior of an extinct carnivore. *Quaternary International* 243(1), 61-79 (2011).
- Palmqvist, P., Ros-Montoya, S., Espigares, M.P., Guerra-Merchán, A., García-Aguilar, J.M., Jiménez-Arenas, J.M., Madurell-Malapiera, J. & Martínez-Navarro, B. Venta Micena: Un yacimiento paleontológico excepcional in *Orce: Homínidos Hienas, Mamuts y Otras Bestias* (eds. Martínez-Navarro, B., Sala, Robert.) 151-188 (Junta de Andalucía, 2018).
- Palmqvist, P., Arribas, A. & Martínez-Navarro, B. Ecomorphological study of large canids from the Early Pleistocene of southeastern Spain. *Lethaia* 32, 75‐88 (1999).
- Palmqvist, P., Gröcke, D. R., Arribas, A & Fariña, R. Paleoecological reconstruction of a Early Pleistocene large mammal community using biogeochemical (δ13C, δ15N, δ18O, Sr:Zn) and ecomorphological approaches. *Paleobiology* 29(2), 205-229 (2003).
- Palmqvist, P., Martínez‐Navarro, B. & Arribas, A. Prey selection by terrestrial carnivores in a Early Pleistocene paleocommunity. *Paleobiology* 22, 514‐534 (1996).
- Palmqvist, P., Torregrosa, V., Pérez-Claros, J. A., Martínez-Navarro, B., & Turner, A. A re-evaluation of the diversity of *Megantereon* (Mammalia, Carnivora, Machairodontinae) and the problem of species identification in extinct carnivores. *Journal of Vertebrate Paleontology* 27(1), 160-175 (2007).
- Pfeiffer, T. *Dama* (*Pseudodama*) *reichenaiui* (Kahlke, 1996) (Artyodactyla, Cervidae, Cervini) aus den Mosbach-Sanden (Wiesbaden-Biebrich). *Mainzer Naturwissenschaftliches Archiv* 35, 31-59 (1997).
- Pfeiffer, T. The first complete skeleton of *Megaloceros verticornis* (Dawkins, 1868) Cervidae, Mammalia, from Bilshausen (Lower Saxony, Germany): description and phylogenetic implications. *Mitteilungen aus dem Museum für Naturkunde Berlin (Geowissenschaftliche Reihe)* 5, 289–308 (2002).
- Pfeiffer, T. The fossil fallow deer *Dama geiselana* (Cervidae, Mammalia, upgrade to species level) in the context of migration and local extinctions of fallow deer in the Late and Middle Pleistocene in Europe. *Paläontologische Zeitschrift* 92, 681-713 (2018).
- Pfeiffer, T. The position of *Dama* (Cervidae, Mammalia) in the system of fossil and living deer from Europe—Phylogenetical analysis based on the postcranial skeleton in *Les Ongulés Holarctiques du Pliocène* (ed. E. Crégut-Bonnoure) 39–57 (Quaternaire, Hors-série 2, 2005).
- Pons-Moyà, J. Los carnívoros (Mammalia) de Venta Micena (Granada, España). *Paleontologia i Evolució* Memoria Especial 1, 109-127 (1987).
- Rodríguez-Gomez, G., Palmqvist, P., Ros-Montoya, S. Espigares, M.P. & Martinez-Navarro, B. Resource availability and competition intensity in the carnivore guild of the Early Pleistocene site of Venta Micena (Orce, Baza Basin, SE Spain). *Quaternary Science Reviews* 164,154-167 (2017).
- Ros‐Montoya, S. Los Proboscídeos del Plio‐Pleistoceno de las cuencas de Guadix‐Baza y Granada. (*Tesis Doctoral, Universidad de Granada*, 2010).
- Ros-Montoya, S., Madurell-Malapiera, J., Martínez-Navarro, B., Espigares, Mª. P. & Palmqvist, P. Late Villafranchian Mammuthus meridionalis (Nesti, 1825) from the Iberian Peninsula: Dentognathic remains from Incarcal-I (Crespià, Girona) and Venta Micena (Orce, Granada). *Quaternary International* 276-277, 17-22 (2012).
- Turner, A. *Megantereon cultridens* (Cuvier) (Mammalia, Felidae, Machairodontinae) from Plio-Pleistocene deposits in Africa and Eurasia, with comments on dispersal and the possibility of a new world origin. *Journal of Paleontology* 61(6),1256-1268 (1987).
- Turner, A. & Antón, M. The giant hyaena, *Pachycrocuta brevirostris* (Mammalia, Carnivora, Hyaenidae). *Geobios.* 29(4), 455-468 (1996).
- Wang, X., Li, Q. & Xie, G. Earliest records of *Sinicuon* in Zanda Basin, Southern Tibet and implications for hypercarnivores in cold climate. *Quaternary International* 355, 3-10 (2015).
- Zrzavý, J., Duda, P., Robovský, J., Okřinová, I. & Pavelková Řičánková, V. Phylogeny of the Caninae (Carnivora): Combining morphology, behaviour, genes and fossils. *Zoologica Scripta* 47(4), 373-389 (2018).

**Supplementary Table S3.** Caption of species and relation to animal size

Very Small size (0) for species less than 25 kg of weight; Small Size (1), including species weighing 25-50 kg; (2), including species weighing 50-125 kg; Intermediate size (3), including species weighing 125-500 kg, with a subdivision of 3a (125-250 kg) and 3b (250-500 kg); (4), including species weighing 500-1,000 kg; and very large species (5), weighing >1,000 kg. Carnivores were classified according to three groups; small carnivores (Size 1) (eg. fox); intermediate carnivore (Size 2) (eg. wolf); and large carnivores (Size 3) (eg. lion).

|  | **Animal Size** |
| --- | --- |
| Herbivore indet. size 0 | <25 kg |
| Herbivore indet. size 1 | 25-50 kg |
| Herbivore indet. size 2 | 50-125 kg |
| Herbivore indet. size 3 | 125-500 kg |
| Herbivore indet. size 3a | 125-250 kg |
| Herbivore indet. size 3b | 250-500 kg |
| Herbivore indet. size 4 | 500-1000 kg |
| Herbivore indet. size 5 | >1,000 kg. |
| *Mammuthus meridionalis* | 5 |
| *Stephanorhinus etruscus* | 5 |
| *Equus altidens* | 3b |
| *Equus* sp. | 3 |
| *Hippopotamus antiquus* | 5 |
| *Bison* sp. | 4 |
| *Hemibos* aff. *gracilis* | 3 |
| *Soergelia minor* | 2 |
| *Capra alba (=Hemitragus albus)* | 2 |
| *Praemegaceros* cf. *verticornis* | 4 |
| *Metacervocerus rhenanus* | 2 |
| Lagomorpha | 0 |
|  |  |
| *Vulpes alopecoides* | Carnivore size 1 |
| *Canis mosbachensis* | Carnivore size 1 |
| *Xenocyon (=Lycaon) lycaonoides* | Carnivore size 2 |
| *Pachycrocuta brevirostris* | Carnivore size 3 |
| *Homotherium latidens* | Carnivore size 3 |
| *Megantereon cultridens* | Carnivore size 3 |
| *Panthera* cf*. gombaszoegensis* | Carnivore size 3 |
| *Ursus etruscus* | Carnivore size 3 |

**Supplementary Tables S4. Skeletal profiles tables.**

|  | **0** | **1** | **2** | **%** | **3** | **%** | **3a** | **%** | **3b** | **%** | **Total 3** | **%** | **5** | **Carniv 1** | **Carniv 2** | **Carniv 3** | **indet** | **Total** | **%^4^** |
| --- | --- | --- | --- | --- | --- | --- | --- | --- | --- | --- | --- | --- | --- | --- | --- | --- | --- | --- | --- |
| Horn |  |  | 1 | 0.7 |  | 0.0 |  | 0.0 |  | 0.0 | 0 | 0.0 |  |  |  |  |  | 1 | 0.2 |
| Cranial |  |  | 1 | 0.7 |  | 0.0 |  | 0.0 |  | 0.0 | 0 | 0.0 |  |  | 1 |  | 2 | 4 | 0.6 |
| Maxilla |  |  |  | 0.0 |  | 0.0 | 1 | 0.9 | 5 | 1.4 | 6 | 1.0 |  |  |  | 2 |  | 8 | 1.3 |
| Mandible |  |  | 9 | 6.4 | 1 | 1.0 | 21 | 17.9 | 6 | 1.6 | 28 | 4.8 |  | 1 | 3 |  |  | 41 | 6.5 |
| Tooth | 1 | 1 | 56 | 40.0 | 6 | 6.0 | 33 | 28.2 | 80 | 21.7 | 119 | 20.3 | 3 | 7 | 22 | 24 | 1 | 234 | 36.9 |
| Vertebra |  |  | 4 | 2.9 | 22 | 22.0 | 2 | 1.7 | 20 | 5.4 | 44 | 7.5 |  |  |  |  | 8 | 56 | 8.8 |
| Rib | 1 |  | 6 | 4.3 | 21 | 21.0 | 1 | 0.9 | 2 | 0.5 | 24 | 4.1 | 1 |  |  |  | 4 | 36 | 5.7 |
| Scapula |  |  | 1 | 0.7 |  | 0.0 |  | 0.0 | 2 | 0.5 | 2 | 0.3 |  |  |  |  |  | 3 | 0.5 |
| Humerus |  |  | 5 | 3.6 |  | 0.0 | 2 | 1.7 | 11 | 3.0 | 13 | 2.2 | 2 | 1 |  |  |  | 21 | 3.3 |
| Radius |  |  | 3 | 2.1 |  | 0.0 | 6 | 5.1 | 6 | 1.6 | 12 | 2.1 | 1 |  |  |  | 1 | 17 | 2.7 |
| Ulna |  |  | 3 | 2.1 |  | 0.0 |  | 0.0 | 8 | 2.2 | 8 | 1.4 | 16 |  | 1 |  |  | 28 | 4.4 |
| Carpal |  |  | 1 | 0.7 |  | 0.0 |  | 0.0 | 2 | 0.5 | 2 | 0.3 |  |  |  |  | 1 | 4 | 0.6 |
| Metacarpal |  |  |  | 0.0 |  | 0.0 | 2 | 1.7 | 15 | 4.1 | 17 | 2.9 |  | 1 | 2 |  |  | 20 | 3.1 |
| Pelvis |  | 1 | 1 | 0.7 | 6 | 6.0 |  | 0.0 | 19 | 5.2 | 25 | 4.3 |  |  | 1 |  |  | 28 | 4.4 |
| Femur | 1 |  | 1 | 0.7 |  | 0.0 | 4 | 3.4 | 7 | 1.9 | 11 | 1.9 |  |  |  |  |  | 13 | 2.0 |
| Patella |  |  |  | 0.0 |  | 0.0 |  | 0.0 |  | 0.0 | 0 | 0.0 | 1 |  |  |  |  | 1 | 0.2 |
| Tibia | 1 |  |  | 0.0 |  | 0.0 | 2 | 1.7 | 30 | 8.2 | 32 | 5.5 |  |  | 4 |  |  | 37 | 5.8 |
| Tarsal | 2 |  | 2 | 1.4 |  | 0.0 | 1 | 0.9 | 10 | 2.7 | 11 | 1.9 |  |  | 1 |  |  | 16 | 2.5 |
| Metapodial | 1 |  | 3 | 2.1 | 2 | 2.0 | 4 | 3.4 | 8 | 2.2 | 14 | 2.4 |  | 1 | 2 |  |  | 21 | 3.3 |
| Metatarsal |  |  | 3 | 2.1 |  | 0.0 | 2 | 1.7 | 18 | 4.9 | 20 | 3.4 |  |  |  |  | 1 | 24 | 3.8 |
| Phalanx | 3 |  | 1 | 0.7 |  | 0.0 |  | 0.0 | 3 | 0.8 | 3 | 0.5 |  | 2 | 6 |  |  | 15 | 2.4 |
| Sesamoid |  |  |  | 0.0 |  | 0.0 |  | 0.0 | 1 | 0.3 | 1 | 0.2 |  |  | 1 |  |  | 2 | 0.3 |
| Turtle Shell | 5 |  |  | 0.0 |  | 0.0 |  | 0.0 |  | 0.0 | 0 | 0.0 |  |  |  |  |  | 5 | 0.8 |
| Indet. | 19 | 1 | 39 | 27.9 | 42 | 42.0 | 36 | 30.8 | 115 | 31.3 | 193 | 33.0 | 4 |  | 3 |  | 715 | 973 |  |
| **Total** | 33 | 3 | 140 | 100.0 | 100 | 100.0 | 117 | 100.0 | 368 | 100.0 | 585 | 100.0 | 28 | 13 | 47 | 26 | 733 | 1608 |  |
|  |  |  |  |  |  |  |  |  |  |  |  |  |  |  |  |  |  |  |  |
| **Cranial^1^** | 1 | 1 | 67 | 66.3 | 7 | 12.1 | 55 | 67.9 | 91 | 36.0 | 153 | 39.0 | 3 | 8 | 26 | 26 | 3 | 288 | 35.7 |
| **Only Cranial^2^** | 0 | 0 | 11 | 10.9 | 1 | 1.7 | 22 | 27.2 | 11 | 4.3 | 34 | 8.7 | 0 | 1 | 4 | 2 | 2 | 54 | 6.7 |
| **Vertebrae and Ribs** | 1 | 0 | 10 | 9.9 | 43 | 74.1 | 3 | 3.7 | 22 | 8.7 | 68 | 17.3 | 1 | 0 | 0 | 0 | 12 | 92 | 11.4 |
| **Cintures** | 0 | 1 | 2 | 2.0 | 6 | 10.3 | 0 | 0.0 | 21 | 8.3 | 27 | 6.9 | 0 | 0 | 1 | 0 | 0 | 31 | 3.8 |
| **Total Axial^3^** | 1 | 1 | 12 | 11.9 | 49 | 84.5 | 3 | 3.7 | 43 | 17.0 | 95 | 24.2 | 1 | 0 | 1 | 0 | 12 | 123 | 15.2 |
| **Upper Limbs**  **(Stylopodium)** | 1 | 0 | 6 | 5.9 | 0 | 0.0 | 6 | 7.4 | 18 | 7.1 | 24 | 6.1 | 2 | 1 | 0 | 0 | 0 | 34 | 4.2 |
| **Intermediate Limbs**  **(Zeugopodium)** | 1 | 0 | 6 | 5.9 | 0 | 0.0 | 8 | 9.9 | 44 | 17.4 | 52 | 13.3 | 18 | 0 | 5 | 0 | 1 | 83 | 10.3 |
| **Lower Limbs**  **(Autopodium)** | 1 | 0 | 6 | 5.9 | 2 | 3.4 | 8 | 9.9 | 41 | 16.2 | 51 | 13.0 | 0 | 2 | 4 | 0 | 1 | 65 | 8.1 |
| **Podials and Phalanges**  **(Autopodium)** | 5 | 0 | 4 | 4.0 | 0 | 0.0 | 1 | 1.2 | 16 | 6.3 | 17 | 4.3 | 0 | 2 | 8 | 0 | 1 | 37 | 4.6 |
|  |  |  |  |  |  |  |  |  |  |  |  |  |  |  |  |  |  |  |  |
| **Anterior Limbs** | 0 | 0 | 13 | 65 | 0 | 0 | 10 | 52.63 | 44 | 34.38 | 54 | 35.29 | 19 | 2 | 3 | 0 | 2 | 93 | 43.9 |
| **Posterior Limbs** | 4 | 1 | 7 | 35 | 6 | 100 | 9 | 47.37 | 84 | 65.6 | 99 | 64.71 | 1 | 0 | 6 | 0 | 1 | 119 | 56.1 |

**Table S4a.** Skeletal Profiles from Venta Micena 4. ^1^All cranial. ^2^All cranial excluded teeth. ^3^Total axial: cintures+ribs+vertebrae. ^4^Excluded indeterminated (Indet.) specimens.

| **Row labels** | **1** | **2** | **%** | **3** | **%** | **3a** | **%** | **3b** | **%** | **Total 3** | **%** | **5** |
| --- | --- | --- | --- | --- | --- | --- | --- | --- | --- | --- | --- | --- |
| **Horn** |  | 1 | 1.0 |  | 0.0 |  | 0.0 |  | 0.0 | 0 | 0.0 |  |
| **Cranial** |  | 1 | 1.0 |  | 0.0 |  | 0.0 |  | 0.0 | 0 | 0.0 |  |
| **Maxilla** |  |  | 0.0 |  | 0.0 | 1 | 1.2 | 5 | 2.0 | 6 | 1.5 |  |
| **Mandible** |  | 9 | 8.9 | 1 | 1.7 | 21 | 25.9 | 6 | 2.4 | 28 | 7.1 |  |
| **Tooth** | 1 | 56 | 55.4 | 6 | 10.3 | 33 | 40.7 | 80 | 31.6 | 119 | 30.4 | 3 |
| **Vertebra** |  | 4 | 4.0 | 22 | 37.9 | 2 | 2.5 | 20 | 7.9 | 44 | 11.2 |  |
| **Rib** |  | 6 | 5.9 | 21 | 36.2 | 1 | 1.2 | 2 | 0.8 | 24 | 6.1 | 1 |
| **Scapula** |  | 1 | 1.0 |  | 0.0 |  | 0.0 | 2 | 0.8 | 2 | 0.5 |  |
| **Humerus** |  | 5 | 5.0 |  | 0.0 | 2 | 2.5 | 11 | 4.3 | 13 | 3.3 | 2 |
| **Radius** |  | 3 | 3.0 |  | 0.0 | 6 | 7.4 | 6 | 2.4 | 12 | 3.1 | 1 |
| **Ulna** |  | 3 | 3.0 |  | 0.0 |  | 0.0 | 8 | 3.2 | 8 | 2.0 | 16 |
| **Carpal** |  | 1 | 1.0 |  | 0.0 |  | 0.0 | 2 | 0.8 | 2 | 0.5 |  |
| **Metacarpal** |  |  | 0.0 |  | 0.0 | 2 | 2.5 | 15 | 5.9 | 17 | 4.3 |  |
| **Pelvis** | 1 | 1 | 1.0 | 6 | 10.3 |  | 0.0 | 19 | 7.5 | 25 | 6.4 |  |
| **Femur** |  | 1 | 1.0 |  | 0.0 | 4 | 4.9 | 7 | 2.8 | 11 | 2.8 |  |
| **Patella** |  |  | 0.0 |  | 0.0 |  | 0.0 |  | 0.0 | 0 | 0.0 | 1 |
| **Tibia** |  |  | 0.0 |  | 0.0 | 2 | 2.5 | 30 | 11.9 | 32 | 8.2 |  |
| **Tarsal** |  | 2 | 2.0 |  | 0.0 | 1 | 1.2 | 10 | 4.0 | 11 | 2.8 |  |
| **Metapodial** |  | 3 | 3.0 | 2 | 3.4 | 4 | 4.9 | 8 | 3.2 | 14 | 3.6 |  |
| **Metatarsal** |  | 3 | 3.0 |  | 0.0 | 2 | 2.5 | 18 | 7.1 | 20 | 5.1 |  |
| **Phalanx** |  | 1 | 1.0 |  | 0.0 |  | 0.0 | 3 | 1.2 | 3 | 0.8 |  |
| **Sesamoid** |  |  | 0.0 |  | 0.0 |  | 0.0 | 1 | 0.4 | 1 | 0.3 |  |
| **Turtle Shell** |  |  | 0.0 |  | 0.0 |  | 0.0 |  | 0.0 | 0 | 0.0 |  |
| **Indet** |  |  | 0.0 |  | 0.0 |  | 0.0 |  | 0.0 |  | 0.0 |  |
| **Total** | 2 | 101 | 100.0 | 58 | 100.0 | 81 | 100.0 | 253 | 100.0 | 392 | 100.0 | 24 |
|  | 1 | 2 | % | 3 | % | 3a | % | 3b | % | Total 3 | % | 5 |
| **Cranial** | 1 | 67 | 66.3 | 7 | 12.1 | 55 | 67.9 | 91 | 36.0 | 153 | 39.0 | 3 |
| **Only Cranial** | 0 | 11 | 10.9 | 1 | 1.7 | 22 | 27.2 | 11 | 4.3 | 34 | 8.7 | 0 |
| **Vertebrae and Ribs** | 0 | 10 | 9.9 | 43 | 74.1 | 3 | 3.7 | 22 | 8.7 | 68 | 17.3 | 1 |
| **Cintures** | 1 | 2 | 2.0 | 6 | 10.3 | 0 | 0.0 | 21 | 8.3 | 27 | 6.9 | 0 |
| **Total Axial** | 1 | 12 | 11.9 | 49 | 84.5 | 3 | 3.7 | 43 | 17.0 | 95 | 24.2 | 1 |
| **Upper Limbs** | 0 | 6 | 5.9 | 0 | 0.0 | 6 | 7.4 | 18 | 7.1 | 24 | 6.1 | 2 |
| **Intermediate Limbs** | 0 | 6 | 5.9 | 0 | 0.0 | 8 | 9.9 | 44 | 17.4 | 52 | 13.3 | 18 |
| **Lower Limbs** | 0 | 6 | 5.9 | 2 | 3.4 | 8 | 9.9 | 41 | 16.2 | 51 | 13.0 | 0 |
| **Podials and Phalanges** | 0 | 4 | 4.0 | 0 | 0.0 | 1 | 1.2 | 16 | 6.3 | 17 | 4.3 | 0 |
|  |  |  |  |  |  |  |  |  |  |  |  |  |
| **Anterior Limbs** | 0 | 13 | 65.0 | 0 | 0.0 | 10 | 52.6 | 44 | 34.4 | 54 | 35.3 | 19 |
| **Posterior Limbs** | 1 | 7 | 35.0 | 6 | 100.0 | 9 | 47.4 | 84 | 65.6 | 99 | 64.7 | 1 |

**Table S4b.** Skeletal Profiles from Venta Micena 4. Excluded indeterminate (Indet.) carnivores and size 0.

**Supplementary Methods. Spatial Analysis.**

Spatial analysis of Venta Micena 4 consisted of three primary analyses, firstly testing for trends on a palaeostratigraphic level across the z-axis, followed by analyses of horizontal distributions across the x and y axes, finishing with a final assessment of preferential orientation patterns.

Supplementary Methods 1. Fossiliferous level detection

For the detection of discrete fossiliferous levels within the vertebrate deposits of Unit C, an artificially intelligent system was employed following the methods proposed by Martín-Perea *et al*. (2020). This system begins by performing pattern recognition via Unsupervised Machine Learning (UML) techniques on 3D spatial coordinates. For UML, a non-parametric Density-Based Spatial Clustering of Applications with Noise (DBSCAN) algorithm was used (Ester *et al*., 1996). DBSCAN accepts two main hyperparameters including a ε value, defining the neighbourhood, followed by a MinPts value which delineates the minimum number of points required to form a cluster. MinPts is generally defined by intuition, with a recommended 2 x nº dimensions rule (Schubert *et al*., 2017), which can then be adjusted depending on the complexity of point distributions and the analyst’s knowledge of the domain under study. The present study found MinPts 3 to be optimal. ε values can then be defined empirically in accordance with this value, using *k*-distance based optimization according to nearest neighbours. To find the optimal ε value, *k*-distance graphs were thus plotted and a *Knee* or *Elbow* point detection algorithm was used to extract the optimal value (Satopää *et al*., 2011).

Results of DBSCAN were then carefully evaluated and studied using a collective Expert-in-the-Loop (EitL) approach (Martín-Perea *et al*., 2020). This process consists in a thorough inspection for any geological features or anomalies that the DBSCAN algorithm may not be able to detect. After this inspection, all points classed as noise by both DBSCAN and the EitL specialists are separated for later classification, while all clearly defined clusters are separated for the final fine-tuning of the spatial stratigraphic profile.

Once spatial points have been passed through both DBSCAN and EitL inspection, the final stage of analyses consist in the fine tuning of layers via Supervised Machine Learning (SML). For SML two algorithms were used, including a Random Forest (RF) and Support Vector Machine (SVM) algorithm. RFs are a robust and highly complex algorithm comprised of multiple decision trees, using a majority vote system to produce the final output (Breiman, 2001). SVMs on the other hand use kernel transformation functions to map out input vectors into a non-linear high dimensional feature space, using soft hyperplanes with maximized margins to calculate the degree of separation between samples (Cortes & Vapnik, 1995). Both algorithms were trained using a *k-*fold cross validation approach (*k* = 10) on 70% of the data for training, while evaluation was performed on the remaining 30%. Additionally, for hyperparameter optimization a random search algorithm was implemented into the training process using 30 iterations to find optimal hyperparameter values.

Original training of SML algorithms revealed both algorithms to have 99% accuracy when used to classify test sets, alongside sensitivity and specificity values of >0.98. These algorithms were then used to classify all points considered indeterminable or noise by both UML and EitL, producing a final fine-tuned profile for the entire site. Classifications were performed with both SVM and RF, taking note of when algorithms came to the same conclusion of disagreed on the class label. When disagreement occurred, the algorithm with the highest confidence making predictions was used for the final classification. For each of the classifications performed, fossils were thus assigned with a probability of association value, using 80% as a threshold to consider a fossil confidently associated to a level or still indeterminable. Finally to assess the quality of the overall system when combining SVM and RF, Cohen’s Kappa coefficient was calculated to determine inter-rater reliability (Cohen, 1960).

Contrary to Martín-Perea *et al*. (2020), the present study did not require the separation of the site into slices in order to detect and fine tune levels. For all analyses the entirety of VM4 was used for fossiliferous level detection.

All Machine Learning applications were performed in the programming language R (v.3.5.1, 64x bit).

Supplementary Methods 2. Spatial Statistics

Once fossiliferous levels had been defined and fine-tuned, spatial point patterns were analysed for each of the levels separately.

Firstly, intensity and concentration of spatial points were visually analyzed through the calculation of density kernels using non-parametric estimation functions. To fine tune these estimates, Diggle’s correction formula was employed (Diggle, 1985), using mean-square error minimization calculations to choose the optimal bandwidth. To quantify the nature of point-pattern intensities, analyses were carried out to test for Complete Spatial Randomness (CSR). CSR, otherwise known as a homogeneous Poisson point process, is a random spatial distribution whereby points within a spatial window are considered independent and homogeneous (Baddeley *et al*., 2016). When using CSR for hypothesis testing, the null hypothesis assumes points to have no preference for any spatial location, while the data obtained from one region of the spatial window is to have no influence on the data obtained from another. CSR can thus be used to test for homogeneity and point pattern intensities numerically via a dispersion test based on quadrat counts. The present analysis performed these tests using an 8 x 6 grid, analogous with the grid system used to excavate the site. Each excavated square is thus defined as a 1m^2^ quadrat where comparisons can be performed to check for contextual CSR. For these tests, Monte Carlo simulations (x10,000) were performed for the calculation of *p*-values, while maps were computed for each of the squares in the site to assess Pearson residual counts (Baddeley *et al*., 2016).

Once CSR had been established, the site was subjected to analyses of spatial correlation in point patterns according to Ripley’s *K*-function and Besag’s *L*-function (Ripley, 1977, Besag, 1977). To adjust for possible weight produced by homogeneity and independence across the spatial window, when CSR was confirmed the traditional function proposed by both Ripley and Besag were used, when CSR was rejected a corrected transformation was accounted for according to Baddeley *et al*. (2000). In either case, these tests plot and compare a theoretical point process function with the observed data to check for correlations. If the corresponding empirical curve is observed to be above the theoretical function, we can conclude that clustering processes are involved. Curves falling below the theoretical function indicate regular processes, while CSR would be confirmed if the two lines coincide. Besag’s *L*-function is a centered adaptation of Ripley’s *K*, typically used to correct any bias that can sometimes impede the interpretation of these graphs. For both tests, necessary transformations were performed for edge and border corrections.

In accordance with spatial correlation tests, complementary hypothesis testing was also performed using the Hopkins-Skellam index (*A*). Hopkins-Skellam tests assess elements of CSR by assessing nearest-neighbour distances and empty-space distances across the window (Hopkins, 1954: with appendix by J.G. Skellam). Values of *A* = 1 typically represent CSR, while *A* < 1 indicates clustering and *A* > 1 describes a regular point pattern. Upon employing Monte Carlo simulations (x10,000) *p*-values can also be calculated.

Upon the statistical detection of cluster point patterns, UML using the DBSCAN algorithm was used to specify the precise location of these clusters (see Notes S4.1). Here MinPts values of 10 were found optimal while ε values were defined using *k*-distance based optimization.

All statistical applications were performed in the programming language R (v.3.5.1, 64x bit).

Supplementary Methods 3. Orientation Patterns

When possible, orientation and azimuth values were taken on site and recorded in degrees (°), while converted into radians (radians = degrees x 180/π) for consequent statistical analyses in R. Circular data and statistics hold particular trigonometric properties that condition all consequent analyses. Under this premise, prior to all analyses statistical tests were performed to determine the nature of the circular distributions present. Orientation patterns from both VM4-I and VM4-II were therefore compared with the Wrapped Normal (Mardia and Jupp, 1999), Cardioid (Jeffreys, 1948), Wrapped Cauchy (McCullagh, 1996), and von Mises distributions (Mardia and Jupp, 1999), using the Mardia-Watson-Wheeler test. Final results concluded that the orientation patterns significantly fitted a von Mises distribution (*W­_g ­_*= 74, *p* = 7.2e-17).

Descriptive statistics were then defined through calculating standardised kurtosis ($\hat{k}$) and skewness ($\hat{s}$) values (Mardia, 1972), followed by sample circular variance (*v*) values, all of which can be derived from the sample mean resultant length of the first trigonometric moment. Both $\hat{k}$ and $\hat{s}$ are interpreted similarly to their linear counterparts, while *v* can be interpreted as the relative concentration of information on a certain part of the circular spectrum; *v* ≈ 0 indicating high concentrations. Standardised skewness is a valuable variable to assess the symmetry of a distribution which, similarly to robust linear statistics (Höhle and Höhle, 2009), can be used to assess the precision of mean values when used for central tendency. In cases where standardised skewness is reported high, mean values are likely to present larger margins of error, requiring the use of more robust metrics such as the median. Under this premise an additional test for symmetry was performed using the second trigonometric moment around the sample mean direction. This test uses the calculation of the test statistic *z*, whereby large values of *z* indicate a significantly skewed distribution (Pewsey, 2002). For the calculation of *p*-values, bootstrap (x10,000) procedures were employed for robustness.

Once the degree of symmetry has been defined, sample mean ($\bar{\theta}$) and median ($\tilde{\theta}$) directions can be calculated to describe the overall central tendency for orientation patterns.

For diagnosing a preferential orientation across the site, and thus defining a degree of anisotropy within the samples, a bootstrapped version of the Rayleigh test was performed with the specified central tendencies calculated for each individual sample (Watson and Williams, 1956).

While resampling methods have been reported to improve the accuracy of statistical inference on circular data (Watson, 1983), to ensure that comparisons between clusters could remain as reliable as possible, a minimum sample size of 25 was enforced used for each of the tests. All clusters, therefore, under n = 25 were therefore excluded from the present analysis.

Finally, azimuth values were incorporated into orientation analyses by the creation of stereoplots.

All circular statistics were performed in the programming language R (v.3.5.1, 64x bit).

|  |  |  |  | **Uniformity** | | **Symmetry** | |  | **16-Point Compass Value^2^** |
| --- | --- | --- | --- | --- | --- | --- | --- | --- | --- |
| **Sample (Size)** | $\hat{\boldsymbol{k}}$ | $\hat{\boldsymbol{s}}$ | ***v*** | **t** | ***p*** | **t** | ***p*** | **Central^1,2^** |  |
| **VM4-I (1125)** | **0.639** | **0.045** | **0.546** | **0.454** | **1.9e-101** | **0.821** | **0.41** | **35** | **NE** |
| Cluster 1 (160) | 2.098 | -0.588 | 0.342 | 0.658 | 4.9e-31 | 2.187 | 0.03 | 27* | NNE* |
| Cluster 2 (31) | -0.122 | 0.073 | 0.830 | 0.090 | 0.17 | 0.273 | 0.68 | 303 | NW |
| Cluster 4 (80) | 0.326 | 0.003 | 0.684 | 0.316 | 2.6e-05 | 0.015 | 0.99 | 54 | NE |
| Cluster 5 (49) | 2.068 | -0.124 | 0.326 | 0.674 | 1.1e-11 | 0.239 | 0.81 | 28 | NNE |
| Cluster 6 (105) | 3.249 | -0.574 | 0.259 | 0.740 | 6.6e-26 | 1.400 | 0.16 | 37 | NE |
| Cluster 7 (57) | 0.105 | 0.139 | 0.810 | 0.021 | 0.19 | 0.946 | 0.34 | 329 | NNW |
| Cluster 9 (226) | 0.318 | 0.289 | 0.500 | 0.500 | 2.2e-26 | 2.636 | 0.01 | 74* | ENE* |
| Cluster 10 (132) | 0.420 | -0.166 | 0.575 | 0.425 | 9.9e-13 | 1.226 | 0.22 | 62 | ENE |
| Cluster 11 (26) | 0.390 | 0.458 | 0.590 | 0.409 | 1.4e-03 | 1.328 | 0.18 | 58 | ENE |
| Cluster 12 (75) | -0.199 | 0.200 | 0.700 | 0.298 | 1.1e-04 | 1.052 | 0.29 | 16 | NNE |
| Cluster 13 (26) | 4.146 | -0.557 | 0.256 | 0.744 | 1.2e-08 | 0.690 | 0.49 | 33 | NNE |
| Cluster 14 (39) | 0.419 | 0.323 | 0.601 | 0.399 | 1.7e-04 | 1.314 | 0.19 | 19 | NNE |
| Cluster 15 (42) | 0.004 | -0.089 | 0.740 | 0.259 | 8.6e-03 | 0.588 | 0.56 | 38 | NE |
| Cluster 16 (36) | 6.094 | 0.770 | 0.262 | 0.738 | 1.7e-10 | 1.460 | 0.14 | 20 | NNE |
| **VM4-II (271)** | **-0.228** | **1.175** | **0.432** | **0.568** | **4.6e-39** | **1.417** | **0.16** | **45** | **NE** |
| Cluster 6 (61) | 0.127 | 0.080 | 0.628 | 0.372 | 1.5e-05 | 0.486 | 0.63 | 35 | NE |
| Cluster 7 (55) | 2.675 | -0.506 | 0.377 | 0.623 | 1.8e-11 | 0.934 | 0.35 | 34 | NE |
| Cluster 9 (98) | 2.513 | -0.890 | 0.257 | 0.740 | 2.0e-24 | 2.100 | 0.04 | 90* | ENE* |

**Table S5**. Descriptive data derived from orientations of fossils in Venta Micena (VM) according to level and cluster. $\hat{k}$ = standardized kurtosis, $\hat{s}$ = standardized skew, *v* = Sample circular variance, t = test statistic, *p* = *p*-value.^1^Values reported in degrees (°). ^2^Central tendencies are measured as a mean ($\bar{\theta}$) or a median ($\tilde{\theta}$) depending on whether non-robust or robust statistical measurements were used. Robust measures are marked with an asterisk (*).

**References**

- Baddeley, A., Rubak, E. & Turner, R. *Spatial Point Patterns: Methodology and Applications with R*. (Taylor and Francis, 2016).
- Besag, J. Contributing to the discussion of the paper by Ripley (1977). *Journal of the Royal Statistical Society, Series B*. **39**, 193-195 (1977).
- Breiman, L. Random Forests. *Machine Learning*. **45(1)**, 5-32 (2001).
- Cohen, J. A coefficient of agreement for nominal scales. *Educational and Psychological Measurement* **20(1)**, 37-46 (1960).
- Cortes, C. & Vapnik, V. Support Vector Networks. *Machine Learning* **20**, 273-297 (1995).
- Diggle, P.J. A kernel method for smoothing point process data. *Journal of the Royal Statistical Society, Series C (Applied Statistics)* **34**, 138-147 (1985).
- Ester, M., Kriegel, H.P., Sander, J. & Xu, X. A density-based algorithm for discovering clusters in large spatial databases with noise. *Proceedings of the 2^nd^ International Conference on Knowledge Discovery and Data Mining, München, Germany*, 226-231(1996).

Höhle, J. & Höhle, M. Accuracy assessment of digital elevation models by means of robust statistical methods. *ISPRS Journal of Photogrammetry and Remote Sensing*. **64**, 398-406 (2009).

- Hopkins, B. A new method of determining the type of distribution of plant individuals. *Annals of Botany* **18**, 213-227 (1954).

Jeffreys, H. *Theory of Probability* (Oxford University, 1948).

Mardia, K.V. *Statistics of Directional Data* (Academic Press, 1972).

- Mardia, K.V. & Jupp, P.E. *Directional Statistics* (John Wiley, 1999).
- Martín-Perea, D.M., Courtenay, L.A., Soledad-Domingo, M. & Morales, J. Application of Artificially Intelligent Systems for the Identification of Discrete Fossiliferous Levels. *PeerJ*. **8** (2020)

McCullagh, P. Möobius transformation and Cauchy parameter estimation. *Annals of Statistics* **24**, 787-808 (1996).

Pewsey, A. Testing circular symmetry. *Canadian Journal of Statistics* **30**, 591-600 (2002).

Ripley, B.D. Modelling spatial patterns (with discussion). *Journal of the Royal Statistical Society, Series B* **39**, 172-212 (1977).

- Satopää, V., Albrecht, J., Irwin, D. & Raghavan, B. Finding a “Kneedle” in a Hyastack: Detecting Knee Points in System Behaviour. *31^st^ International Conference on Distributed Computing Systems Workshops,* 166-171 (2011).
- Schubert, E., Sander, J., Ester, M., Kreigel, H.P. & Xu, X. DBSCAN revisited: why and how you should (still) use DBSCAN. *ACM Transactions on Database Systems* **42(3)**, 1-21 (2017).

Watson, G.S. *Statistics on Spheres* (John Wiley, 1983).

Watson, G.S. & Williams, E.J. On the construction of Significance tests on the Circle and on the Sphere. *Biometrika* **43**, 344-352 (1956).
